# PIWI-interacting RNA expression regulates pathogenesis in a *Caenorhabditis elegans* model of Lewy body disease

**DOI:** 10.1038/s41467-023-41881-8

**Published:** 2023-10-02

**Authors:** Xiaobing Huang, Changliang Wang, Tianjiao Zhang, Rongzhen Li, Liang Chen, Ka Lai Leung, Merja Lakso, Qinghua Zhou, Hongjie Zhang, Garry Wong

**Affiliations:** 1 Guangdong Provincial Key Laboratory of Tumor Interventional Diagnosis and Treatment, Zhuhai Institute of Translational Medicine, Zhuhai People’s Hospital Affiliated with Jinan University, Jinan University, Zhuhai, 519000 China; 2 Cancer Centre, Centre of Reproduction, Development and Aging, Faculty of Health Sciences, University of Macau, 999078 Macau, China; 3 GMU-GIBH Joint School of Life Sciences, Guangzhou Laboratory, Guangzhou Medical University, Guangzhou, 510799 China; 4 Department of Computer Science, College of Engineering, Shantou University, Shantou, 515063 China; 5 Department of Anesthesiology, The First Affiliated Hospital, Jinan University, Guangzhou, 510630 China; 6 Biomedical Translational Research Institute, Faculty of Medical Science, Jinan University, Guangzhou, 510632 China

**Keywords:** Diseases of the nervous system, Molecular neuroscience

## Abstract

PIWI-interacting RNAs (piRNAs) are small noncoding RNAs that regulate gene expression, yet their molecular functions in neurobiology are unclear. While investigating neurodegeneration mechanisms using human α-syn(A53T)^Tg^ and Aβ^Tg^;α-syn(A53T)^Tg^ pan-neuronal overexpressing strains, we unexpectedly observed dysregulation of piRNAs. RNAi screening revealed that knock down of piRNA biogenesis genes improved thrashing behavior; further, a *tofu-1* gene deletion ameliorated phenotypic deficits in α-syn(A53T)^Tg^ and Aβ^Tg^;α-syn(A53T)^Tg^ transgenic strains. piRNA expression was extensively downregulated and H3K9me3 marks were decreased after *tofu-1* deletion in α-syn(A53T)^Tg^ and Aβ^Tg^;α-syn(A53T)^Tg^ strains. Dysregulated piRNAs targeted protein degradation genes suggesting that a decrease of piRNA expression leads to an increase of degradation ability in *C. elegans*. Finally, we interrogated piRNA expression in brain samples from PD patients. piRNAs were observed to be widely overexpressed at late motor stage. In this work, our results provide evidence that piRNAs are mediators in pathogenesis of Lewy body diseases and suggest a molecular mechanism for neurodegeneration in these and related disorders.

## Abstract (web summary)

piRNAs are small noncoding RNAs whose biological functions are not fully understood. Here, the authors demonstrate using C. elegans genetic models that dysregulated piRNAs are associated with protein degradation processes in a model of Lewy body disease.

## Introduction

**Fig. 1:**
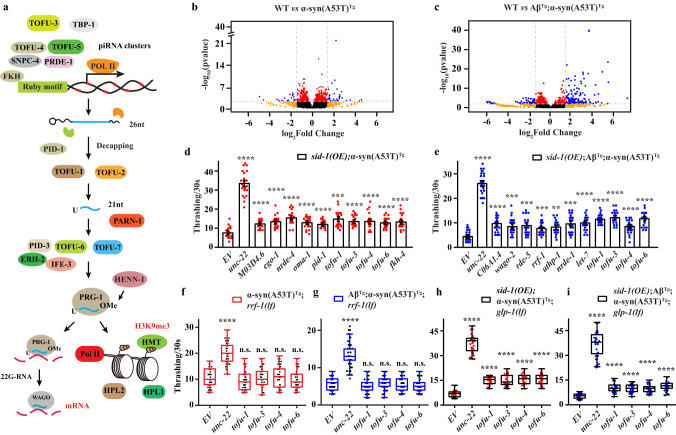
RNAi screening in transgenic animals. **a** Schematic diagram of piRNA biogenesis in *C. elegans*. **b**, **c** Volcano plot of piRNAs expression in small RNA-Seq. Red dots, *P* value < 0.01, FDR < 0.05; orange dots, |log_2_(fold change)| > 1.5; blue dots, *P* value < 0.01, FDR < 0.05 and |log_2_(fold change)| > 1.5; black dots, *P* value ≥ 0.01, FDR ≥ 0.05 and |log_2_(fold change)| ≤ 1.5. **d**, **e** Thrashing assay in young adult stage of *sid-1(OE)*;α-syn(A53T)^Tg^ and *sid-1(OE)*;Aβ^Tg^;α-syn(A53T)^Tg^ strains under different RNAi clones (*n* ≥ 12 for each clone). Data are presented as mean ± SEM. **f**–**i** Thrashing assay in the young adult stage of different strains under *tofu-1*, *tofu-3*, *tofu-4*, and *tofu-6* RNAi clones (*n* ≥ 12 for each clone). The box plots display the median, upper, and lower quartiles; the whiskers show a 1.5× interquartile range (IQR). For (**d**–**i**), each dot represents one nematode. *EV* was an empty vector (negative control), and *unc-22* was a positive control in the RNAi screen. *p*-values were determined by one-way ANOVA analysis. ****P < 0.0001, ***P < 0.001, **P < 0.01, n.s. not significant. Source data are provided as a Source Data file.

piRNAs are small noncoding RNAs derived from single-stranded precursor transcripts expressed in piRNA clusters located in intergenic regions of the genome^1,2^. piRNA biogenesis begins with transcription of primary piRNAs, which are then shortened by RNA exonucleases at both 5’ and 3’ ends with a preference for a 5’ U residue, and are then 3’ nucleotide 2’O-methylated (Fig. 1a). This results in 26–30 nucleotide (nts) piRNAs in *Drosophila* and vertebrates, and 21 nts in *C. elegans* (21U-RNA)^3^. A forward genetic screen for factors involved in *C. elegans* piRNA biogenesis uncovered several *tofu* (Twenty-One-Fouled Ups) genes that greatly perturbed 21U-RNA production^4^. TOFU-1 and TOFU-2 knock down led to precursor 21U (pre-21U) accumulation suggesting that they are involved in downstream processing. TOFU-3,-4,-5 knock down led to a lack of 21U-RNA suggesting they are also required for piRNA precursor transcription. TOFU-6 and TOFU-7 affect mature levels of 21U-RNAs without affecting precursor levels suggesting they are involved in the loading of piRNAs to PRG-1^5^.

Primary piRNAs bind PIWI proteins and undergo secondary amplification known as the “Ping-Pong” cycle in *Drosophila* and mammals^6^. While in *C. elegans*, piRNAs bind with PRG-1, and secondary siRNAs are generated via RNA dependent RNA polymerases (RdRps)^4^. Fully processed and amplified piRNAs bind PIWI proteins to form mature piRNA-PIWI complexes that regulate transposable elements or cellular gene expression^4^. piRNAs repress gene expression mainly through post-transcriptional silencing by inducing 22G-RNA biogenesis and silencing mRNA transcripts directly or initiate transcriptional silencing by inducing chromatin modification, such as repressive histone mark formation or DNA methylation^7,8^. Accumulating evidence suggests that PIWI/piRNAs are broadly expressed in the nervous system and participate in neuronal processes as well as neuronal disorders^9^. The Piwi/piRNA complex promotes serotonin-dependent methylation in the CREB2 promoter in *Aplysia*, which enhances long-term synaptic facilitation^10^. piRNAs target neuronal mRNAs, which regulate dendritic spine morphogenesis^11^. In addition, a neuronal piRNA pathway was found to inhibit axon regeneration in *C. elegans*^12^. piRNA not only regulates memory formation but also induces transgenerational inheritance of learning pathogen avoidance behavior to the offspring in *C. elegans*^13,14^. Loss of condensation of heterochromatin and depletion of piRNAs can drive transposable element dysregulation, which is a driver for tauopathy neurodegeneration^15^. Together, these studies provide an emerging concept of piRNAs as an important regulator in the maintenance and function of neuronal processes.

Amyloid β (Aβ) and α-synuclein (α-syn) represent two of the key proteins found in lesions associated with age-related neurodegenerative disorders such as Alzheimer’s (AD) and Parkinson’s disease (PD), respectively^16^. Parkinson’s disease (PD) and Lewy body Dementia (LBD) are two neurodegenerative disorders related to Lewy body formation and accumulation, which leads to persistent movement deficits and worsens over time^17^. Currently, there is no effective treatment for PD and LBD. Our lab previously produced PD and LBD models in *C. elegans*, which overexpressed human α-syn(A53T) pan-neuronally for the PD model and co-expressed human Aβ and α-syn(A53T) pan-neuronally for the LBD model (transgenic lines of α-syn(A53T)^Tg^ and Aβ^Tg^;α-syn(A53T)^Tg^), respectively^18,19^. Both of these models displayed dopaminergic neuronal loss and motor deficits, which allowed us to explore the pathogenesis of PD and LBD^18,19^.

The role of piRNAs in neurobiology is less well understood. We previously observed that piRNAs were dysregulated in α-syn(A53T)^Tg^ animals as part of a study to characterize changes in the small noncoding RNA landscape during neurodegeneration^20^. In that study, we described our initial observation and did not investigate the mechanism.

In this work, we hypothesize that piRNAs are dysregulated and aggravate neuronal loss in Lewy body diseases. Here we find that piRNAs are dysregulated in α-syn(A53T)^Tg^ and Aβ^Tg^;α-syn(A53T)^Tg^ transgenic animals and are mainly upregulated in these strains. When we knock down piRNA biogenesis genes, we observe an improvement in movement in α-syn(A53T)^Tg^ and Aβ^Tg^;α-syn(A53T)^Tg^ strains, suggesting a role for piRNAs in mediating neurodegeneration. We further cross these model strains with a *tofu-1* null mutant to explore the function of piRNAs in Lewy body related diseases. TOFU-1 loss of function mutation (*lf*) in α-syn(A53T)^Tg^ and Aβ^Tg^;α-syn(A53T)^Tg^ strains ameliorate the behavioral phenotypes and improve thrashing, extend lifespan, and reduce α-syn expression while alleviating the dopaminergic neuron degeneration. piRNAs are broadly downregulated after *tofu-1* deletion. Furthermore, these piRNAs require PRG-1 to regulate thrashing deficits, and the repressive epigenetic marks of H3K9me3 increase in α-syn(A53T)^Tg^ and Aβ^Tg^;α-syn(A53T)^Tg^ strains, while decreasing after *tofu-1* deletion in transgenic animals. piRNA mainly targets protein degradation genes, which lead to α-syn accumulation, whereas decreases of piRNA expression increases the protein degradation ability in *C. elegans*. We further find that piRNAs are aberrantly expressed in PD patient brains and are mainly overexpressed in the late motor stage, suggesting that piRNA dysregulation might be conserved from *C. elegans* to humans. We thus provide insights to explain the roles of piRNAs in neurodegenerative diseases related with protein degradation, suggesting that piRNAs are one of the key components in mediating neuropathology in Lewy body diseases. These findings indicate a pathway to explore treatment and biomarker development for neuropathology involved in Lewy body diseases.

## Results

### piRNAs dysregulation in α-syn(A53T)^Tg^ and Aβ^Tg^;α-syn(A53T)^Tg^ strains

In order to explore how small RNAs regulate pathogenesis in neurodegenerative diseases, we used small RNA-Seq to identify changes of small RNAs in our PD and LBD models, which overexpressed human α-syn(A53T) and Aβ;α-syn(A53T) pan-neuronally in *C. elegans*. To our surprise, we found that piRNAs were dysregulated both in α-syn(A53T)^Tg^ and Aβ^Tg^;α-syn(A53T)^Tg^ transgenic strains and some of the piRNAs were overexpressed in these two models. For the α-syn(A53T)^Tg^ transgenic strain, 34 differentially expressed piRNAs (DEPs, |log_2_fold change | >1.5, *P* < 0.01 and FDR < 0.05) were identified with 23 DEPs upregulated and 11 DEPs downregulated (Fig. 1b, Supplementary Data 1). These 34 DEPs target 1131 genes. For the Aβ^Tg^;α-syn(A53T)^Tg^ strain, 117 DEPs were identified with 94 DEPs upregulated and only 23 DEPs downregulated (Fig. 1c, Supplementary Data 1). These 117 DEPs target 2338 genes. Three piRNAs (*21ur-10824, 21ur-11898, 21ur-13215*) were both upregulated in these two models. We used qRT-PCR to confirm some of the piRNA expression changes and these results were consistent with our small RNA-Seq (Supplementary Fig. 1a, b). Previous studies observed that piRNA expression changes in neurodegenerative diseases^9^. A study identified massive dysregulated piRNAs in PD derived neuronal cells^21^. AD patients showed that 103 piRNAs were nominally differentially expressed in the brains and among this change, 81 piRNAs were upregulated and 22 piRNAs were downregulated^22^. Similarly, another study identified 146 piRNAs that were upregulated and only 3 piRNAs that were downregulated in AD patients^23^. Based on our sequencing results and these previous data from patients, we speculated that piRNAs were dysregulated in Lewy body diseases.

We hypothesized that if piRNAs were dysregulated and mainly overexpressed in neurodegenerative diseases, a decrease in piRNA expression might improve phenotypes observed in neurodegenerative disease models. To confirm our hypothesis, we used RNAi to knock down piRNA biogenesis genes and determined whether it can improve thrashing in α-syn(A53T)^Tg^ and Aβ^Tg^;α-syn(A53T)^Tg^ transgenic strains. Neurons are highly resistant to exogenous dsRNA in *C. elegans*, therefore, we overexpressed *sid-1(OE)* in α-syn(A53T)^Tg^ and Aβ^Tg^;α-syn(A53T)^Tg^ strains by standard genetic crossing to increase RNAi efficiency^24^. The biogenesis of type I piRNAs is initiated by fork head (FKH) family transcription factors that recognize the Ruby motif^4,9^. The 26nt of 21U-RNA precursors generated are exported to cytoplasm, then further modified by TOFU-1, TOFU-2, TOFU-6, TOFU-7 to generate the mature 21U-RNAs in *C. elegans* (Fig. 1a). The mature 21U-RNAs combine with PRG-1 to form RNA induced silencing complexes (RISC) and induced 22G-RNAs biogenesis to target mRNA directly or cause histone modification to silence gene expression^4,9^. In our study, we screened 130 genes, which covered most of the small RNAs biogenesis genes in our models (Supplementary Data 2). In the first-round of screening, we identified 19 genes that could improve thrashing in *sid-1(OE)*;α-syn(A53T)^Tg^ strains, and 23 genes in *sid-1(OE)*;Aβ^Tg^;α-syn(A53T)^Tg^ strains (*P* < 0.01, thrashing > 1.5 fold) (Supplementary Data 2). Among these genes, we performed a second-round of screening and finally confirmed 10 genes that could significantly improve thrashing in *sid-1(OE)*;α-syn(A53T)^Tg^ strains and 11 genes in *sid-1(OE)*;Aβ^Tg^;α-syn(A53T)^Tg^ transgenic strains (*P* < 0.01, thrashing > 1.5 fold) (Fig. 1d, e). Among these genes, we found that piRNA biogenesis genes *tofu-1*, *tofu-3*, *tofu-4*, and *tofu-6* can improve thrashing both in *sid-1(OE)*;α-syn(A53T)^Tg^ and *sid-1(OE)*; Aβ^Tg^;α-syn(A53T)^Tg^ transgenic strains (Fig. 1d, e). Furthermore, we assayed these genes on locomotion in *sid-1(OE)*;α-syn(A53T)^Tg^ and *sid-1(OE)*;Aβ^Tg^;α-syn(A53T)^Tg^ strains. The results confirmed that knock down of *tofu-1*, *tofu-3*, *tofu-4*, and *tofu-6* improved movement in our models (Supplementary Fig. 1c, d), suggesting that the piRNAs might be involved in the pathogenesis of Lewy body diseases.

piRNAs were originally discovered in germline cells and act to silence transposable elements, while more recent accumulating evidence suggests that piRNAs are expressed in somatic cells and may participate in neuronal functions^9–12^. In order to address whether piRNAs improved thrashing behavior independent of germline function, we used tissue specific RNAi in α-syn(A53T)^Tg^;*rrf-1(lf)* and Aβ^Tg^;α-syn(A53T)^Tg^;*rrf-1(lf)* transgenic strains. Strains with a *rrf-1* null mutation are sensitive to RNAi against genes expressed in the germline (some in intestine and hypodermal cells), and *rrf-1* loss of function mutation is widely used in RNAi to assess the function of genes specifically in the germline in *C. elegans*^25^. Our results showed that knock down of *tofu-1*, *tofu-3*, *tofu-4*, and *tofu-6* did not improve thrashing in α-syn(A53T)^Tg^;*rrf-1(lf)* and Aβ^Tg^;α-syn(A53T)^Tg^;*rrf-1(lf)* transgenic animals (Fig. 1f, g). We further crossed *sid-1(OE)*;α-syn(A53T)^Tg^ and *sid-1(OE)*; Aβ^Tg^;α-syn(A53T)^Tg^ strains with the temperature sensitive strain *glp-1(e2141)III*, which will lose germline cells after *C. elegans* are cultivated in 23 °C. We found that knock down of *tofu-1*, *tofu-3*, *tofu-4*, and *tofu-6* could still improve thrashing in *sid-1(OE)*;α-syn(A53T)^Tg^;*glp-1(lf)* and *sid-1(OE)*;Aβ^Tg^;α-syn(A53T)^Tg^;*glp-1(lf)* in 23°C (Fig. 1h, i). Since piRNAs required PRG-1 to form RISC and previous work showed that *prg-1* mRNA is present in somatic cells^12^; therefore, our results suggested that piRNAs may improve thrashing in somatic cells, which would be independent of germline function.

### TOFU-1 loss of function mutation ameliorates behavioral impairments in α-syn(A53T)^Tg^ and Aβ^Tg^;α-syn(A53T)^Tg^ strains

**Fig. 2:**
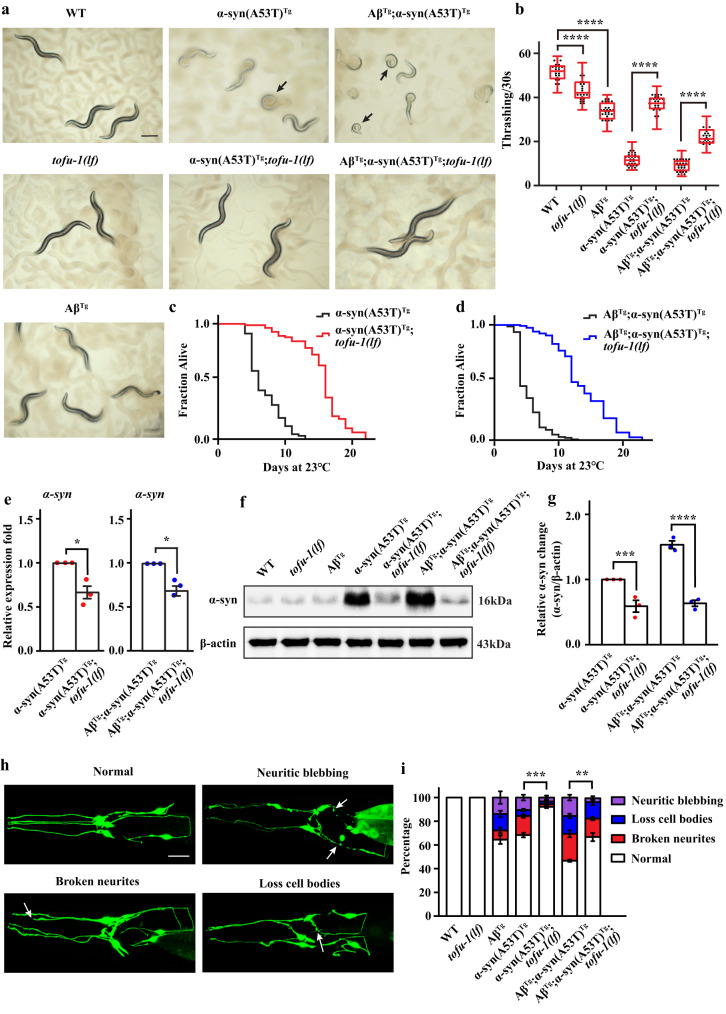
TOFU-1 deletion ameliorates behavioral impairments in transgenic animals. **a** Transmitted light microscopy images of transgenic animals on agar plates. Black arrows indicate transgenic animals with severe postural deficits. Scale bar = 250 μM. **b** Thrashing assay of WT and transgenic animals at young adult stage (*n* ≥ 12 for each group). The box plots display the median, upper, and lower quartiles; the whiskers show 1.5× interquartile range (IQR). **c**, **d** The survival curve of transgenic animals (*n* ≥ 70 for each group). The average lifespan was analyzed by Kaplan–Meier test, *P* value was calculated by the log-rank test. **e** Gene expression of α-syn in transgenic animals (mean ± SEM, *n* = 3). **f**, **g** Western blot analysis of α-syn expression in transgenic animals at young adult stage (mean ± SEM, *n* = 3). **h** Confocal imaging of normal, neuritic blebbing, broken neurites, and loss cell body in head dopaminergic neurons in transgenic animals. Typical examples are shown. White arrows indicate the location of the lesion. Images were obtained using Zeiss confocal microscope LSM710 at 400× magnification. Scale bar = 20 μM. **i** Quantitation of dopaminergic neuron lesions on day 8 adults (*n* ≥ 60 for each group). Statistical analysis was conducted for the normal ratio in dopaminergic neurons in transgenic strains, three biological replicates (mean ± SEM). For (**b**, **g**, **i**), *p*-values were determined by one-way ANOVA analysis. For (**e**), *p*-values were determined by two-tail student’s *t-*test. ****P < 0.0001, ***P < 0.001, **P < 0.01, **P < 0.001, *P < 0.05. Source data are provided as a Source Data file.

To further confirm that piRNAs are involved in pathogenesis, we focused on TOFU-1. TOFU-1 (21U-RNA biogenesis fouled up) gene was originally identified from forward genetic screens directed towards piRNA biogenesis and function^5^. It is enriched in neurons and germline in *C. elegans*^5^. Since knock down of *tofu-1* improves thrashing and locomotion in our transgenic animals, we crossed *tofu-1* loss of function mutant (strain *tm6424*) to α-syn(A53T)^Tg^ and Aβ^Tg^;α-syn(A53T)^Tg^ strains to generate the compound transgenic animal α-syn(A53T)^Tg^;*tofu-1(lf)* and Aβ^Tg^;α-syn(A53T)^Tg^;*tofu-1(lf)*. Our results showed that *tofu-1* deletion visibly reversed the coiled postures into sinuous shapes both in α-syn(A53T)^Tg^ and Aβ^Tg^;α-syn(A53T)^Tg^ transgenic strains (Fig. 2a). We analyzed images and the results showed that 70.7% ± 1.41 of α-syn(A53T)^Tg^, 84.2% ± 1.25 of Aβ^Tg^;α-syn(A53T)^Tg^, while 24.8% ± 1.29 of α-syn(A53T)^Tg^;*tofu-1(lf)*, and 35.9% ± 3.48 of Aβ^Tg^;α-syn(A53T)^Tg^;*tofu-1(lf)* animals (mean ± SEM) showed postural deficits. We then assayed behavioral phenotypes and showed that compared with α-syn(A53T)^Tg^ and Aβ; α-syn(A53T)^Tg^ strains, α-syn(A53T)^Tg^;*tofu-1(lf)* and Aβ^Tg^;α-syn(A53T)^Tg^;*tofu-1(lf)* transgenic animals had significantly improved thrashing rates (Fig. 2b). We also assayed effects on lifespan and observed that *tofu-1* loss of function mutation significantly extended lifespan of α-syn(A53T)^Tg^ and Aβ^Tg^;α-syn(A53T)^Tg^ strains (Fig. 2c, d). Compared with the wild type, *tofu-1(lf)* mutant also extended lifespan in *C. elegans* (Supplementary Fig. 2a). Meanwhile, we also checked whether *tofu-1* loss of function mutation can improve phenotypes in pan-neuronal expression of Aβ transgenic animal (strain of CL2355). Our results showed that *tofu-1(lf)* mutation improved learning ability, increased thrashing rate, and decreased Aβ gene expression in the Aβ^Tg^ strain (Supplementary Fig. 2b–d), suggesting that *tofu-1* loss of function reversed phenotypes in neurodegenerative disease models in *C. elegans*.

α-Syn can be observed as protein deposits in PD, while both Aβ and α-syn accumulate in LBD to cause neurodegeneration^26,27^. Since Aβ did not change while α-syn increases dramatically in LBD models in *C. elegans*^19^, we mainly focused on how piRNAs regulate α-syn levels in α-syn(A53T)^Tg^ and Aβ^Tg^;α-syn(A53T)^Tg^ strains. We wondered whether *tofu-1* deletion reduced α-syn expression. Our results showed that α-syn was decreased both in mRNA and protein levels in α-syn(A53T)^Tg^;*tofu-1(lf)* and Aβ^Tg^;α-syn(A53T)^Tg^;*tofu-1(lf)* transgenic strains (Fig. 2e–g). Since monomer α-syn further aggregates into multimer forms, we used native gel electrophoresis to test the different forms of α-syn in transgenic strains. The results showed that *tofu-1* deletion not only decreased the monomer but also the polymer form of α-syn in α-syn(A53T)^Tg^;*tofu-1(lf)* and Aβ^Tg^;α-syn(A53T)^Tg^;*tofu-1(lf)* transgenic strains (Supplementary Fig. 2e). We also assayed Aβ gene expression in the Aβ^Tg^;α-syn(A53T)^Tg^ strain. Our results showed that Aβ levels decreased after *tofu-1* deletion (Supplementary Fig. 2f).

Previous studies showed that both α-syn(A53T)^Tg^ and Aβ^Tg^;α-syn(A53T)^Tg^ strains had degeneration of dopaminergic neurons, which was mainly caused by α-syn overexpression and accumulation^18,19^. We further determined whether *tofu-1* loss of function mutation improved neurodegeneration in α-syn(A53T)^Tg^ and Aβ^Tg^;α-syn(A53T)^Tg^ strains. Aberrant neurons characterized by broken neurites, shrinking of dendritic endings, neuritic blebbing, and the loss of neuronal cell bodies could be observed (Fig. 2h)^28^. We observed the neurodegeneration in day 8 adults, while some of the animals were dead in α-syn(A53T)^Tg^ and Aβ^Tg^;α-syn(A53T)^Tg^, therefore, these surviving animals might have better neuronal morphology than the population average in α-syn(A53T)^Tg^ and Aβ^Tg^;α-syn(A53T)^Tg^ strains. Our results showed that after *tofu-1* deletion, dopaminergic neurons showed 92.2% normal morphology in day 8 adults compared to 68.3% normal morphology in the α-syn(A53T)^Tg^ strain, and 66.7% normal morphology in Aβ^Tg^;α-syn(A53T)^Tg^;*tofu-1(lf)* transgenic animals compared to only 46.6% normal morphology in Aβ^Tg^;α-syn(A53T)^Tg^ strains (Fig. 2i). These results suggest that *tofu-1* loss of function mutation ameliorated some of the phenotypic deficits and alleviated the dopaminergic neuron degeneration in α-syn(A53T)^Tg^ and Aβ^Tg^;α-syn(A53T)^Tg^ transgenic animals.

### TOFU-1 loss of function mutation downregulates piRNAs expression

In order to determine whether *tofu-1* deletion decreases piRNAs, we used small RNA-Seq to measure piRNA expression in α-syn(A53T)^Tg^;*tofu-1(lf)* and Aβ^Tg^;α-syn(A53T)^Tg^;*tofu-1(lf)* strains. Compared with wild type, piRNAs were dramatically downregulated in α-syn(A53T)^Tg^;*tofu-1(lf)* and Aβ^Tg^;α-syn(A53T)^Tg^;*tofu-1(lf)* transgenic animals. In α-syn(A53T)^Tg^;*tofu-1(lf)*, 2078 DEPs were downregulated while only 83 DEPs were upregulated (|log_2_fold change | >1.5, *P* < 0.01 and FDR < 0.05) (Supplementary Fig. 3a and Supplementary Data 1). For Aβ^Tg^;α-syn(A53T)^Tg^;*tofu-1(lf)* strain, 1598 DEPs were downregulated, and 55 DEPs were upregulated (Supplementary Fig. 3a and Supplementary Data 1). These results showed that *tofu-1* deletion significantly downregulated piRNA expression in α-syn(A53T)^Tg^ and Aβ^Tg^;α-syn(A53T)^Tg^ transgenic strains. Since *tofu-1* is one of the piRNA biogenesis genes, we also measured piRNA expression in *tofu-1(lf)* mutant. Results confirmed that *tofu-1* deletion significantly downregulated piRNAs expression with 1913 DEPs downregulated and only 88 DEPs upregulated (Supplementary Fig. 3a and Supplementary Data 1).

We made a further comparison between α-syn(A53T)^Tg^ and α-syn(A53T)^Tg^;*tofu-1(lf)* strains, Aβ^Tg^;α-syn(A53T)^Tg^ and Aβ^Tg^;α-syn(A53T)^Tg^;*tofu-1(lf)* strains. Our results confirmed that *tofu-1* loss of function mutation significantly downregulated piRNA expression. In α-syn(A53T)^Tg^;*tofu-1(lf)* strain, 1967 DEPs were downregulated, and 88 DEPs were upregulated compared to α-syn(A53T)^Tg^ transgenic animals (Supplementary Fig. 3a and Supplementary Data 1). For Aβ^Tg^;α-syn(A53T)^Tg^;*tofu-1(lf)* strain, 971 DEPs were downregulated and only 27 DEPs were upregulated compared to Aβ^Tg^;α-syn(A53T)^Tg^ strain (Supplementary Fig. 3a and Supplementary Data 1). We found one piRNA (*21ur-3554*) that was upregulated in WT *vs*. α-syn(A53T)^Tg^ and downregulated in WT *vs*. α-syn(A53T)^Tg^;*tofu-1(lf)*, and three piRNAs (*21ur-6799, 21ur-8461, 21ur-7000*) that were upregulated piRNAs in WT *vs*. Aβ^Tg^;α-syn(A53T)^Tg^ and downregulated in WT *vs*. Aβ^Tg^;α-syn(A53T)^Tg^;*tofu-1(lf)*.

TOFU-1 is involved in piRNA processing by enhancing the conversion of the precursor 21U (pre-21U, 26nt) into mature piRNA (21U-RNA, 21nt) as silencing *tofu-1* or loss of *tofu-1* function increases the pre-21U levels of piRNA in *C. elegans*^5^. To explore whether the downregulation of piRNAs after *tofu-1* deletion in our transgenic models is due to the pre-21U accumulation, we analyzed the length distribution in these transgenic strains. Results confirmed that the pre-21U extensively accumulated in *tofu-1* deletion strains (Supplementary Fig. 3b, c), suggesting that the downregulation of piRNAs was mainly due to the precursor piRNA accumulation. Combined with experiments that showed behavioral phenotype deficits were rescued and piRNAs were downregulated in α-syn(A53T)^Tg^;*tofu-1(lf)* and Aβ^Tg^;α-syn(A53T)^Tg^;*tofu-1(lf)* strains, our results suggest that downregulation or inhibition of piRNA expression may decrease misfolded protein expression in α-syn(A53T)^Tg^ and Aβ^Tg^;α-syn(A53T)^Tg^ transgenic animals.

### H3K9me3 levels increase in α-syn(A53T)^Tg^ and Aβ^Tg^;α-syn(A53T)^Tg^ strains while decreasing after *tofu-1* loss of function mutation

piRNAs regulate gene expression mainly through post-transcriptional or/and transcriptional silencing, these two processes required PRG-1 to form RISC^2,29,30^. Previous research detected a significant amount of *prg-1* mRNA in germline less *C. elegans* and confirmed their presence in somatic cells using single-molecule fluorescence in situ hybridization^12^. In order to determine whether piRNAs recruit PRG-1 to mediate thrashing deficits, we crossed the *prg-1* loss of function mutant to the overexpressing transgenic animals to generate compound strains α-syn(A53T)^Tg^;*prg-1(lf)*. PRG-1 loss of function still improved thrashing in α-syn(A53T)^Tg^;*prg-1(lf)* strains (Supplementary Fig. 4a). Furthermore, we used RNAi to knock down *tofu-1* in *sid-1(OE)*;α-syn(A53T)^Tg^;*prg-1(lf)* strains with the result showing that *tofu-1* could not improve thrashing (Supplementary Fig. 4b), suggesting that TOFU-1 required PRG-1 to execute its function.

**Fig. 3:**
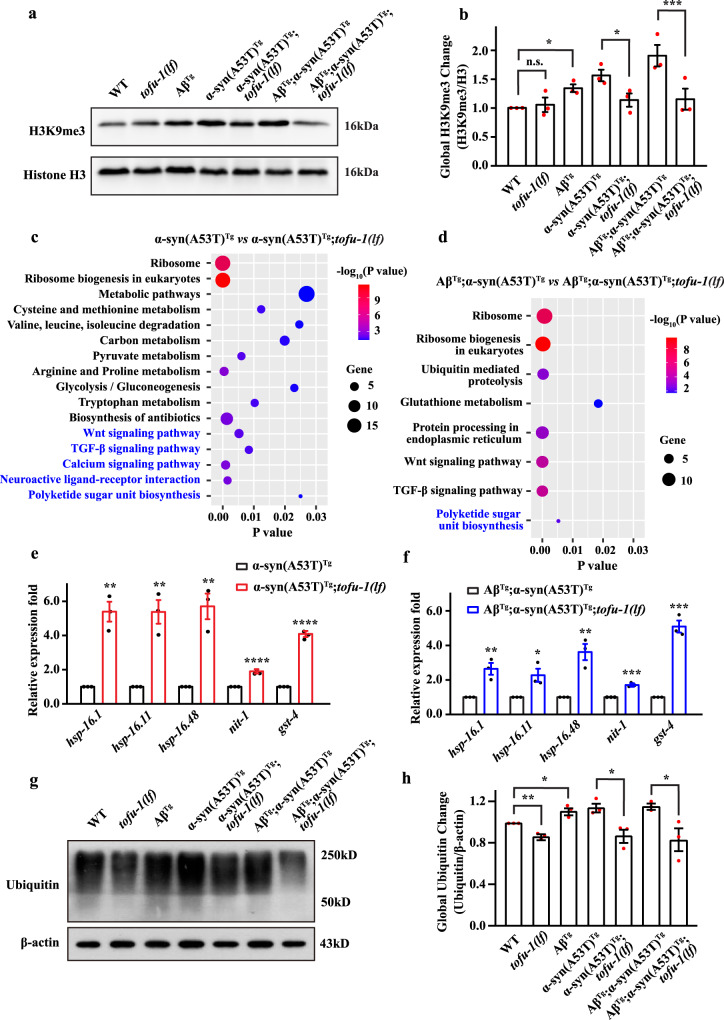
H3K9me3 change and RNA-Seq analysis in transgenic animals. **a**, **b** Western blot analysis of H3K9me3 expression in transgenic animals at young adult stage (mean ± SEM, *n* = 3). **c**, **d** Enriched KEGG pathways analysis in transgenic animals. Pathways are shown on the left side of the chart. Black text indicates upregulated pathways and blue text indicates downregulated. The size of the balls denotes the number of genes in pathways that are regulated. −log_10_ (*P* value) is indicated by the color of the circle and the *P* value is indicated by the location on the horizontal axis. **e**, **f** Gene expression in transgenic animals (mean ± SEM, *n* = 3). **g**, **h** Western blot analysis of ubiquitination in transgenic animals in young adult stages (mean ± SEM, *n* = 3). For (**b**, **h**), *p*-values were determined by one-way ANOVA analysis. For (**e**, **f**) *p*-values were determined by two-tailed student’s *t-*test. ****P < 0.0001, ***P < 0.001, **P < 0.01, **P < 0.001, *P < 0.05, n.s. not significant. Source data are provided as a Source Data file.

piRNAs are loaded to PRG-1 to form RISC complexes and recruit histone marks of H3K9me3 to silence gene expression^30^. Thus, we examined whether H3K9me3 marks were changed in α-syn(A53T)^Tg^ and Aβ^Tg^;α-syn(A53T)^Tg^ transgenic animals in genetic backgrounds that included or excluded the *tofu-1* gene. Our results showed that the levels of H3K9me3 increased in α-syn(A53T)^Tg^ and Aβ^Tg^;α-syn(A53T)^Tg^ strains compared with wild type, while H3K9me3 levels decreased in α-syn(A53T)^Tg^;*tofu-1(lf)* and Aβ^Tg^;α-syn(A53T)^Tg^;*tofu-1(lf)* compared with our transgenic models (Fig. 3a, b). We further crossed H3K9me3 methyltransferase null mutant genes *set-32* and *set-25* into *sid-1(OE)*;α-syn(A53T)^Tg^ and *sid-1(OE)*;Aβ^Tg^;α-syn(A53T)^Tg^ strains. SET-32 and SET-25 are required for the establishment of epigenetic silencing signals in *C. elegans*^6^. Our results showed that knock down of *tofu-1* could not improve thrashing in *sid-1(OE)*;α-syn(A53T)^Tg^;*set-32(lf)*, *sid-1(OE)*;α-syn(A53T)^Tg^;*set-25(lf)*, *sid-1(OE)*;Aβ^Tg^;α-syn(A53T)^Tg^;*set-32(lf)* and *sid-1(OE)*;Aβ^Tg^;α-syn(A53T)^Tg^;*set-25(lf)* transgenic strains (Supplementary Fig. 4d–g), suggesting that the piRNAs required repressive epigenetic marks of H3K9me3 to regulated gene expression, while downregulation of piRNA expression decreases H3K9me3 levels.

### Expression of protein degradation genes decrease in α-syn(A53T)^Tg^ and Aβ^Tg^;α-syn(A53T)^Tg^ strains while increase after *tofu-1* loss of function mutation

Since repressive epigenetic marks of H3K9me3 increased in α-syn(A53T)^Tg^ and Aβ^Tg^;α-syn(A53T)^Tg^ strains while being decreased after *tofu-1* deletion, we carried out ChIP-Seq to explore the H3K9me3 modification. Because piRNAs are mostly overexpressed in α-syn(A53T)^Tg^ and Aβ^Tg^;α-syn(A53T)^Tg^ strains, we mainly investigated the function of these overexpressed piRNAs in neurodegenerative diseases. After annotating modified peaks, we identified 116 and 146 differentially H3K9me3 modified genes (|log_2_fold change | >2 and *P* < 0.05) in α-syn(A53T)^Tg^ and Aβ^Tg^;α-syn(A53T)^Tg^ strains, respectively compared with wild type in ChIP-Seq (Supplementary Fig. 5a and Supplementary Data 3). ChIP-qPCR further confirmed the alteration of some modified genes in α-syn(A53T)^Tg^ and Aβ^Tg^;α-syn(A53T)^Tg^ strains (Supplementary Fig. 5b, c). GO enrichment analysis showed that piRNAs mainly inhibited translation and ribosome function in α-syn(A53T)^Tg^ and Aβ^Tg^;α-syn(A53T)^Tg^ strains, suggesting that overexpression of piRNAs in our transgenic model primarily targeting genes involved in protein synthesis (Supplementary Fig. 5d, e).

piRNAs can silence transcripts through imperfectly complementary sites by initiating small interfering RNA response^29,30^, therefore, we analyzed the 22G-RNA expression. Our results suggested that 22G-RNAs showed the same expression trend with piRNAs. 22G-RNA expression levels increased in α-syn(A53T)^Tg^ and Aβ^Tg^;α-syn(A53T)^Tg^ strains, while decreased after *tofu-1* deletion (Supplementary Data 4). We further predicted the target genes for piRNAs by using piRTarBase^31^. For WT *vs* α-syn(A53T)^Tg^, 23 overexpressed piRNAs target 935 genes, and 4 genes overlapped with 259 downregulated differentially expressed genes (DEGs) in RNA-Seq (Supplementary Fig. 6a)^19^. Among these 4 overlapped genes, *T21E12.3* is predicted to be involved in proteolysis, which was downregulated in the α-syn(A53T)^Tg^ strain. RNAi knock down of *T21E12.3* slightly decreased thrashing in *sid-1(OE)*;α-syn(A53T)^Tg^ strain (Supplementary Fig. 6e). For WT *vs* Aβ^Tg^;α-syn(A53T)^Tg^, 94 overexpressed piRNAs target to 1838 genes. Of these, 54 genes overlapped with 605 downregulated DEGs in RNA-Seq (Supplementary Fig. 6c). Among these overlapped genes, *C08H9.1* and *R03G8.6* are predicted to regulate proteolysis activity, while decreased in Aβ^Tg^;α-syn(A53T)^Tg^ strain, and RNAi showed that decrease of these two genes exacerbated thrashing in *sid-1(OE)*;Aβ^Tg^;α-syn(A53T)^Tg^ strain (Supplementary Fig. 6f). Further enrichment analysis found that some of these upregulated piRNAs targets were enriched in endocytosis, phagosome, and MAPK signaling pathways, which are related with the protein degradation both in α-syn(A53T)^Tg^ and Aβ^Tg^;α-syn(A53T)^Tg^ strains (Supplementary Fig. 7a, b)^32^.

We further carried out RNA-Seq to compare gene expression before and after *tofu-1* loss of function mutation in α-syn(A53T)^Tg^ and Aβ^Tg^;α-syn(A53T)^Tg^ strains. TOFU-1 deletion mainly increased the ribosome biogenesis, metabolic process, ubiquitin mediated proteolysis, and protein processing in endoplasmic reticulum ontologies, suggesting that the degradation ability increased after *tofu-1* deletion in α-syn(A53T)^Tg^;*tofu-1(lf)* and Aβ^Tg^;α-syn(A53T)^Tg^;*tofu-1(lf)* transgenic strains (Fig. 3c, d and Supplementary Data 5). Some protein folding genes like the heat shock proteins (*hsp-16.1, hsp-16.11, hsp-16.48*), which enable unfolded protein binding activity; *nit-1* (enable hydrolase activity) and *gst-4* (enables glutathione transferase activity) genes are all upregulated in α-syn(A53T)^Tg^;*tofu-1(lf)* and Aβ^Tg^;α-syn(A53T)^Tg^;*tofu-1(lf)* strains compared with α-syn(A53T)^Tg^ and Aβ^Tg^;α-syn(A53T)^Tg^ transgenic animals both in RNA-Seq and qRT-PCR (Fig. 3e, f). Western blot further showed that ubiquitination increased in α-syn(A53T)^Tg^ and Aβ^Tg^;α-syn(A53T)^Tg^ strains, while decreased after *tofu-1* deletion (Fig. 3g, h).

Taken together, these results suggest that overexpressed piRNAs target protein folding and degradation both in transcriptional and post-transcriptional gene silencing in α-syn(A53T)^Tg^ and Aβ^Tg^;α-syn(A53T)^Tg^ strains; while *tofu-1* deletion increased protein degradation genes expression in α-syn(A53T)^Tg^;*tofu-1(lf)* and Aβ^Tg^;α-syn(A53T)^Tg^;*tofu-1(lf)* transgenic animals.

### Lysosome function increases in α-syn(A53T)^Tg^;*tofu-1(lf)* and Aβ^Tg^;α-syn(A53T)^Tg^;*tofu-1(lf)* transgenic strains

Since the sequencing data showed that piRNAs mainly target protein degradation genes, we further investigated lysosome activity to confirm whether protein degradation function was improved after *tofu-1* deletion in our models based on several rationale. Firstly, our previous results showed that lysosome function mainly decreased in Aβ^Tg^;α-syn(A53T)^Tg^ strains and in one type of LBD patient, dementia with Lewy body (DLB)^19^. Clinically, post-mortem PD brains showed the depletion of intraneuronal lysosomes, accumulation of undegraded autophagosomes, and reduction in the lysosome related proteins^33^. Lysosomal dysfunction is central to PD and other neurodegenerative diseases^34^. Secondly, protein degradation occurs through autophagy-lysosome and ubiquitin-proteasome pathways^35^; while the lysosome is at the crossroads of various degradative pathways including endocytosis and autophagy to regulate exogenous and endogenous cellular molecules^36^. Thirdly, α-syn processing is lysosomal-dependent, which affects its turnover, accumulation, and aggregation^37^. For instance, the mutation of glucocerebrocidase (GBA), which encodes a lysosomal enzyme involved in sphingolipid degradation, is the single greatest risk factor based on genome-wide association studies (GWAS) in PD and LBD^38,39^.

**Fig. 4:**
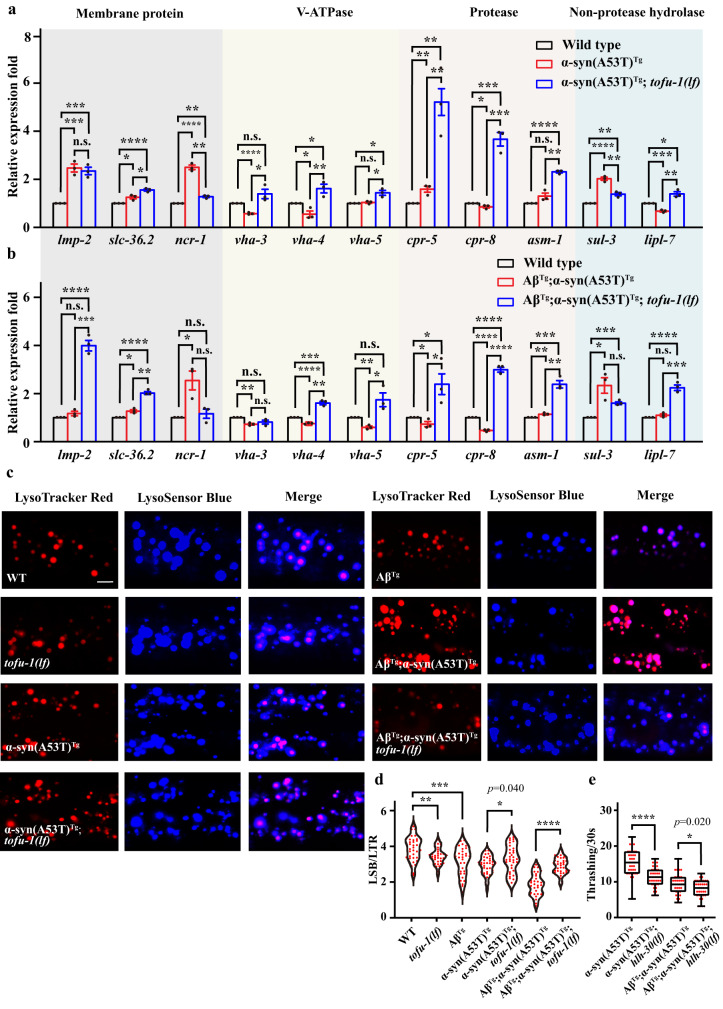
Lysosome function in transgenic animals. **a**, **b** qRT-PCR analysis of lysosomal gene expression in wild type and transgenic animals. The bottom of the bar chart indicates the gene name. The top of the bar chart indicates the type of gene. Bars are color coded to indicate different transgenic strains (mean ± SEM, *n* = 3). **c** Confocal imaging of lysosome morphology and acidity in transgenic animals by lysotracker red (LTR) and lysosensor blue (LSB) staining. Typical examples for each strain are shown. Images were obtained using Zeiss confocal microscope LSM710 at 630× magnification. Scale bar = 5 μM. **d** The fluorescence intensity of LSB/LTR in each strain was quantified. Each dot represents one nematode (*n* ≥ 30 for each group). **e** Thrashing assay in transgenic strains at young adult stage. The box plots display the median, upper, and lower quartiles; the whiskers show 1.5× interquartile range (IQR). Each dot represents one nematode (*n* ≥ 12 for each group). For (**a**, **b**, **d**, **e**), *p*-values were determined by one-way ANOVA analysis. ****P < 0.0001, ***P < 0.001, **P < 0.01, *P < 0.05. n.s. not significant. Source data are provided as a Source Data file.

Based on this background and combined with the protein degradation genes increase after *tofu-1* loss of function mutation, we expected to observe that the overexpressed piRNAs would lead to decreased lysosome related gene expression levels in α-syn(A53T)^Tg^ and Aβ^Tg^;α-syn(A53T)^Tg^ strains while increasing in α-syn(A53T)^Tg^;*tofu-1(lf)* and Aβ^Tg^;α-syn(A53T)^Tg^;*tofu-1(lf)* transgenic animals. We used qRT-PCR to verify some of lysosomal gene expression in *C. elegans*. These genes encode lysosomal membrane proteins of *lmp-2, slc-36.2, ncr-1*, the proton pump V-ATPase of *vha-3, vha-4, vha-5*, the protease of *cpr-5, cpr-8, asm-1*, and the hydrolases of *sul-3, lipl-7* (Fig. 4a, b)^40^. Our results demonstrated that the gene expression levels of proteases *cpr-5, cpr-8* and *asm-1* were significantly increased both in α-syn(A53T)^Tg^;*tofu-1(lf)* and Aβ^Tg^;α-syn(A53T)^Tg^;*tofu-1(lf)* strains compared with α-syn(A53T)^Tg^ and Aβ^Tg^;α-syn(A53T)^Tg^ strains (Fig. 4a, b). Previous results showed that α-syn is primarily degraded by lysosomal proteases and cathepsin D, which is consistent with our results here^41^. Other genes like *vha-3*, *vha-4*, and *lipl-7* were decreased in α-syn(A53T)^Tg^, and *vha-4*, *vha-5* were decreased in Aβ^Tg^;α-syn(A53T)^Tg^, while they recovered after *tofu-1* loss of function mutation in *C. elegans* (Fig. 4a, b). Furthermore, the lysosomal membrane proteins *lmp-2, and slc-36.2* were significantly increased in Aβ^Tg^;α-syn(A53T)^Tg^;*tofu-1(lf)* strains (Fig. 4b).

In order to observe lysosome function at the cellular level, we co-stained with LysoTracker Red (LTR) and LysoSensor Blue (LSB) to monitor the morphology and acidity of the lysosome in transgenic animals. LSB is sensitive to acidity but not to LTR; therefore, LTR is used as a control for normalizing the LSB intake, and the fluorescence intensity ratio of LSB/LTR is used to indicate the activity of lysosome acidity^40^. Our results showed that lysosome acidity was dramatically reduced in α-syn(A53T)^Tg^ and Aβ^Tg^;α-syn(A53T)^Tg^ strains, while significantly improved in α-syn(A53T)^Tg^;*tofu-1(lf)* and Aβ^Tg^;α-syn(A53T)^Tg^;*tofu-1(lf)* transgenic animals (Fig. 4c, d), suggesting that lysosome activity is improved after *tofu-1* deletion in α-syn(A53T)^Tg^ and Aβ^Tg^;α-syn(A53T)^Tg^ strains.

The TFEB orthologue HLH-30 is a master transcription factor for autophagy and lysosome biogenesis^42^, we further crossed *hlh-30* mutant with α-syn(A53T)^Tg^ and Aβ^Tg^;α-syn(A53T)^Tg^ strains; results showed that the thrashing was further damaged after *hlh-30* loss of function mutation in our transgenic animals (Fig. 4e).

Taken together, these results strongly suggest that piRNAs target protein degradation and reduce its function, whereas the degradation function was recovered after *tofu-1* loss of function mutation by downregulating piRNAs expression.

### piRNAs are dysregulated in pre-motor early and late motor stages of PD patients

**Fig. 5:**
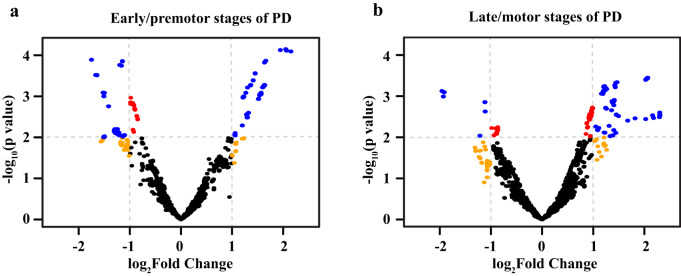
piRNAs expression in PD patients. **a**, **b** Volcano plot of piRNA expression in premotor and motor stages of PD patients. Data were obtained from GEO database (GSE97285) which includes 7 early premotor PD, 7 late motor stage PD, and 14 control samples. Red dots, *P* value < 0.01; orange dots, |log2(fold change)| > 1; blue dots, *P* value < 0.01 and |log2(fold change)| > 1; black dots, *P* value ≥ 0.01 and |log_2_(fold change)| ≤ 1. Data are provided in Supplementary Data 6.

In order to connect our findings to human disease, we analyzed small RNA-Seq data in PD patients^43^. In pre-motor PD patients, we identified 945 piRNAs that were upregulated and 810 piRNAs that were downregulated (Fig. 5a and Supplementary Data 6); whereas in late-motor PD patients, we found 731 piRNAs were upregulated and 66 piRNAs were downregulated (Fig. 5b and Supplementary Data 6). These results are consistent with our model that piRNAs are dysregulated in Lewy body diseases. In the late-motor stages of PD, most piRNAs were overexpressed, suggesting that a decrease in piRNA expression might be a potential therapeutic strategy for Lewy body diseases.

## Discussion

For our PD model, we expressed human α-syn with alanine53 mutant to threonine (A53T) pan-neuronally under the *aex-3* promoter. For the LBD model, we co-expressed Aβ with α-syn(A53T) (*aex-3* promoter) while Aβ was under the *snb-1* promoter^19^. Since our model expresses these proteins pan-neuronally, our western blots here and in previous publications^18,19^ show a high level of α-syn expression. Since *C. elegans* does not have a known α-syn ortholog and we overexpressed a mutant form of α-syn(A53T) pan-neuronally, our phenotypes may be more severe than other PD models that express α-syn in muscle cells. We did not compare the α-syn expression levels between our model with other PD models since the promoters, variants, and copy numbers of the α-syn transgene differ depending upon the model.

In our models of PD and LBD, which expresses α-syn(A53T) and Aβ;α-syn(A53T) pan- neuronally, we profiled small noncoding RNAs and unexpectedly found piRNA dysregulation characterized by predominant upregulation of piRNAs in transgenic animals, which was consistent with our previous work sequencing the α-syn(A53T)^Tg^ strain^20^. We were then prompted to identify genetic interactors and found that knock down of piRNA biogenesis genes suppressed the impairment phenotypes suggesting that piRNAs mediate neuropathic deficits. Follow-up studies identified *tofu-1*, *tofu-3*, *tofu-4*, and *tofu-6* as suppressors in α-syn(A53T)^Tg^ and Aβ^Tg^;α-syn(A53T)^Tg^ strains. Tissue specific RNAi suggested that the functions of *tofu-1*, *tofu-3*, *tofu-4*, and *tofu-6* in our model were independent of germline function.

While all these genes perturb piRNA biogenesis, we focused on *tofu-1* because it was enriched in neurons, functions late in biogenesis, and null mutants demonstrated strong deficits in the abundance of mature piRNAs^5^. Crosses of null mutant *tofu-1* into single α-syn(A53T)^Tg^, Aβ^Tg^ or double Aβ^Tg^;α-syn(A53T)^Tg^ overexpressing transgenic animals revealed that TOFU-1 was necessary for thrashing, lifespan, and neuron morphology impairments, thus implicating mature piRNAs as mediators of neurodegeneration in our model. These *tofu-1* mutants showed decreased α-syn expression in overexpressing transgenic animals. By using small RNA sequencing, we confirmed that *tofu-1* deletion caused a massive downregulation of piRNAs, and this downregulation was mainly caused by piRNA precursor accumulation in our transgenic animals. Since *tofu-1* is involved in piRNA biogenesis, we first considered that the piRNA pathway was essential to improve phenotypes in our transgenic models. We further observed that both *tofu-1* and *prg-1* mutants had extended lifespan compared with wild type animals, suggesting that *tofu-1* not only functions in the piRNA pathway, but may also participate elsewhere to regulate biological processes to mediate lifespan extension. This suggests that *tofu-1* deletion might improve phenotypes through non-piRNA mediated pathways and thus, we cannot rule out multiple actions of TOFU-1 in *C. elegans*.

We also examined the function of *tofu-1* deletion in another PD model which expresses wild type α-syn in muscle cells (strain NL5901). We did not find that *tofu-1* deletion decreased α-syn expression and aggregation in this α-syn(WT)^Tg^ model (Supplementary Fig. 8a–d). We speculate that *tofu-1* did not express or function in muscle cells, which could not change the α-syn expression in α-syn(WT)^Tg^ strain. Since piRNAs recruit histones to activate or silence gene expression, we hypothesized that piRNAs affect gene expression of α-syn and other targets via epigenetic modification. We first identified repressive epigenetic marks of H3K9me3 increased in α-syn(A53T)^Tg^ and Aβ^Tg^;α-syn(A53T)^Tg^ strains while decreased after *tofu-1* deletion. These results suggested that piRNAs act mainly through repressive epigenetic marks to execute its silencing function. Previous research observed that 4 piRNAs could reciprocally regulate 4 mRNAs (*CYCS, LIN7C, KPNA6, RAB11A*) in AD brain, suggesting that a direct action may be possible^23^. We also wondered whether some individual piRNAs could directly decrease α-syn expression by post-transcription; however, we were unable to identify dysregulated piRNAs that targeted sequences for α-syn transcripts directly, which prompted us to consider whether piRNAs regulated other processes to change the α-syn level.

By using ChIP-Seq, 21U-RNA target prediction, and RNA-Seq, we showed that the function of dysregulated piRNAs was related to protein degradation. ChIP-Seq indicated that overexpressed piRNAs might recruit repressive epigenetic marks of H3K9me3 to silence the transcription and synthesis of protein related genes. 21U-RNA target prediction found that some piRNA targets are directly related to protein folding, which decreased in α-syn(A53T)^Tg^ and Aβ^Tg^;α-syn(A53T)^Tg^ strain. Enrichment analysis for overexpressed piRNA targets also showed that these targets are enriched in folding related pathways. Furthermore, by using RNA-Seq to observe the changes in α-syn(A53T)^Tg^;*tofu-1(lf)* and Aβ^Tg^;α-syn(A53T)^Tg^;*tofu-1(lf)* transgenic animals, we were able to confirm that overexpressed piRNAs target to protein folding genes, which decreases its degradation ability in α-syn(A53T)^Tg^ and Aβ^Tg^;α-syn(A53T)^Tg^ transgenic models, while the degradation activity was restored after deletion of *tofu-1* gene. We tried to find out overlapped genes between ChIP-Seq and RNA-Seq, while we only identified a few overlapped genes. One possible explanation would be that the epigenetic regulation of dysregulated genes is more complex than we anticipate. For example, the use of more and different Chip-Seq antibodies may uncover more overlaps. Another explanation would be that our Chip-Seq or RNA-Seq methods are not sensitive enough to uncover overlapping genes. Finally, the role of post transcriptional regulation may be underestimated in our study.

Since α-syn is mainly degraded by the lysosomal-autophagic pathway and combined with our previous study that showed lysosome function was decreased in an LBD model and patients; and that the mutation of GBA, which encodes a lysosomal enzyme involved in sphingolipid degradation, is the single greatest risk factor for PD and LBD^38,39^; and LBD patients have 6 to 8 times higher risk as a carrier with GBA mutant^44^; we were prompted to further investigate lysosome function. Our results confirmed that lysosome function decreased in α-syn(A53T)^Tg^ and Aβ^Tg^;α-syn(A53T)^Tg^ strains whereas its function improved after *tofu-1* loss of function mutation and piRNA downregulation. Even though our results show that *tofu-1* deletion improves phenotypes, further studies will still need to be conducted to strengthen the relationship between piRNA function and protein degradation. If piRNAs were overexpressed in the transgenic model of α-syn(A53T)^Tg^ and Aβ^Tg^;α-syn(A53T)^Tg^, we would expect to see increased piRNA expression in patient tissues. To test this, we analyzed a publicly available dataset (GSE97285) of small RNA reads from control and early premotor or late motor PD brain tissues^43^. In this dataset, we found both upregulated and downregulated piRNAs (945 up and 810 down) in early pre-motor, but more upregulated piRNAs (731 up and 66 down) in late motor stage samples, entirely consistent with the hypothesis of increased piRNAs in neurodegeneration in Lewy body diseases. The dysregulation of piRNAs was shown in PD neuronal cells and AD patients previously^21–23^; while the function of piRNAs in neurodegenerative diseases are not well understood. By using a PD and LBD model in *C. elegans*, we showed that dysregulated piRNAs targeted protein degradation, and provided a plausible explanation for the roles of piRNAs in neurons. Nonetheless, more animal models and datasets will be needed in the future to expand the knowledge of how small noncoding RNAs function in somatic cells in both healthy and disease states.

Another alternative explanation of how piRNAs exacerbate neurodegenerative processes, besides epigenetic modification, maybe via their traditional targets, transposons. While we did not investigate transposon expression here, it was reported in a *Drosophila* tauopathy model that a differential expression of more than 100 transposable elements could be observed^15^. Furthermore, a study of AD brains showed a high transcriptional activation of transposable elements that correlated with neurofibrillary tangles, a pathological hallmark in AD^45^. While beyond the scope here, future studies will investigate various downstream effects of piRNA dysregulation including their mRNA as well as noncoding RNA targets.

**Fig. 6:**
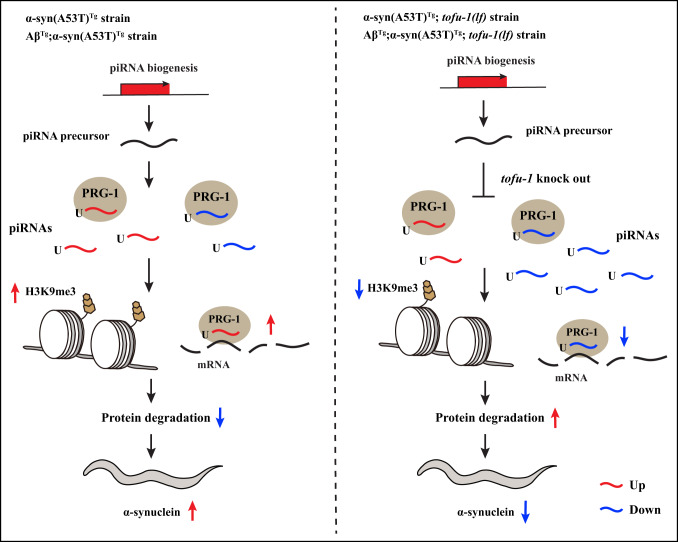
Proposed piRNA function in transgenic animals. In α-syn(A53T)^Tg^ and Aβ^Tg^;α-syn(A53T)^Tg^ strains, piRNA dysregulation upregulates H3K9me3 to silence and/or directly target protein degradation genes which cause α-syn accumulation. After blocking piRNA biogenesis by knocking out the *tofu-1* gene, piRNAs downregulate and decrease H3K9me3, while protein degradation gene expression increases, which improves the activity of protein degradation and decreases α-syn expression in *C. elegans*.

We found piRNAs dysregulated both in α-syn(A53T)^Tg^ and Aβ^Tg^;α-syn(A53T)^Tg^ transgenic animals. Deletion of piRNA biogenesis gene of *tofu-1* ameliorates behavioral phenotypes, improves thrashing, extends lifespan, decreases α-syn expression, and alleviates dopaminergic neuron degeneration in α-syn(A53T)^Tg^ and Aβ^Tg^;α-syn(A53T)^Tg^ strains. The *tofu-1* loss of function mutation extensively downregulated piRNAs, which were mainly caused by piRNA precursor accumulation in our transgenic models. piRNAs required PRG-1 to improve thrashing after *tofu-1* loss of function mutation. Further, repressive epigenetic marks of H3K9me3 were increased in α-syn(A53T)^Tg^ and Aβ^Tg^;α-syn(A53T)^Tg^ transgenic animals, whereas *tofu-1* deletion returned these levels to near wild type strain levels. piRNAs mainly target protein degradation genes, which leads to misfolded protein accumulation through post-transcriptional and transcriptional gene silencing. Downregulated piRNA expression reversed behavioral phenotypes in transgenic animals. Based on the body of our results, we propose a sequence of piRNA actions in α-syn(A53T)^Tg^ and Aβ^Tg^;α-syn(A53T)^Tg^ strains both before and after *tofu-1* gene deletion (Fig. 6), where piRNAs can mediate the neurodegenerative effects of α-syn(A53T)^Tg^ and Aβ^Tg^;α-syn(A53T)^Tg^ strains. Lastly, we found piRNAs were overexpressed in the late stage of PD patients, indicating that these effects might be conserved from *C. elegans* to humans. Our data suggests that piRNAs regulate the pathogenesis of Lewy body diseases, and that reduction of piRNA expression may ameliorate symptoms in Lewy body diseases. This work provides a role of piRNA in neurons and may thus open other avenues to investigate the molecular basis for neurodegenerative diseases.

Some limitations still exist in our work. We were unable to identify specific piRNAs that target Aβ or α-syn directly, which would provide direct evidence about piRNA function in the context of these pathological proteins. In addition, it remains unclear how *tofu-1* regulates lifespan extension, and we still do not understand how this common effect (piRNA pathway and lifespan extension) improves phenotypes in PD and LBD models. Although PRG-1 was detected in somatic cells, it remains to be determined whether PRG-1 is localized in neurons. Furthermore, we are unable to identify piRNA targets in PD patients since most of the piRNA processes are obscure in humans. Knowledge concerning the biogenesis of piRNAs and related processes mainly comes from animal models like mouse, *Drosophila*, and *C. elegans*; therefore, how piRNA biogenesis occurs and the identification of their putative targets in humans deserves further exploration.

## Methods

### Strains and maintenance

All strains were cultured on NGM plates seeded with *Escherichia coli* OP50 at 16 °C unless otherwise indicated. For experiments, *C. elegans* was transferred to 23 °C in order to activate the Aβ peptide expression at L3 stage. New strains were created by standard genetic crosses and genotypes were confirmed by PCR or sequencing. The strains used and created in this project are listed in Supplementary Data 7.

### Thrashing assay

*C. elegans* were cultivated at 16 °C till they reached L3, then transferred to 23 °C until growth to L4 or young adults. Late L4 or young adult nematodes were placed in a drop of M9 buffer and allowed to recover for 30 s, then video recordings of the body bends for 30 s for each strain under the microscope were taken. Some of the recordings were analyzed by Image J software under the plugin of wrMTrck^46^. At least 12 nematodes were counted in each experiment. The experiment was performed 3 times with independent biological replicates.

### Locomotion assay

*C. elegans* were cultivated at 16 °C until they reached L3, then transferred to 23 °C until growth to adults. Day 1 old adult nematodes were chosen randomly from RNAi plates and transferred to fresh plates seeded with OP50. After 5 min recovery period, the number of body bends was counted per minute on each nematode by visual observation on a Nikon SMZ700 dissecting microscope at 60× magnification. At least 12 nematodes were counted for each experiment. Each experiment was performed 3 times with independent biological replicates.

### Lifespan assay

All strains were cultured on NGM plates for 2–3 generations without starvation at 16 °C. For the assay, *C. elegans* were cultivated at 16 °C till they reached L3, then transferred to 23 °C until they grew to L4 or young adult. Late L4 or young adult nematodes were transferred to NGM plates seeded with fresh OP50 and defined as experiment day 0. Animals were transferred to fresh plates every other day. If *C. elegans* did not respond to mechanical stimulation from a platinum wire, they were scored as dead. If *C. elegans* crawled off the plate, displayed extruded internal organs, or died due to the hatching of progeny inside the uterus, these nematodes were censored. The entire lifespan assay was repeated with at least 3 independent trials.

### RNAi plasmid construction

RNAi plasmids for *tofu-3*, *tofu-6* were produced by PCR cloning and ligation into pL4440 vector (Addgene). All primer pairs included *XhoI* and *NheI* restriction sequences (*tofu-3*: 5’- AAAGCTAGCTCCAAAATGCGAAGCACCC; 3’- AAACTCGAGTGCACAATCACGAG GAACCA; *tofu-6*: 5’-AAACTCGAGCCGTGTTGTTGGACAATCC; 3’- AAAGCTAGCGG AAGACTCCATCGTAGCC). *C. elegans* were collected by M9, and total RNA was extracted by using RNAiso Plus (Takara). Leftover DNA was digested with RQ1 RNase-free DNase (Promega). The purified RNA was reverse transcribed to cDNA by RevertAid Reverse Transcriptase (Thermo Fisher Scientific). Two microliters of the RT cDNA products were used in each PCR reaction with Phusion DNA polymerase (Thermo Fisher Scientific) and purified by PCR purification kit (Qiagen). The vector pL4440 and PCR product were digested by *XhoI* and *NheI* enzymes and ligated by T4 DNA ligase (Thermo Fisher Scientific). The ligation reactions were terminated by heat-inactivation and transfected into *DH5a* competent bacteria. Ampicillin resistant bacteria colonies were grown, and their plasmids were isolated and sequenced to verify correct sequences. Finally, the verified plasmids were transfected into *HT115* and further verified by restriction digestion and sequencing again to confirm the correct sequences.

### RNA interference

RNA interference (RNAi) was performed on NGM plates containing 2 mM isopropyl β-D-1-thiogalactopyranoside (IPTG) and 25 μg/mL carbenicillin. RNAi bacteria were cultured in LB liquid (100 μg/mL ampicillin) with each target gene fragment or empty vector (Source from BioScience Nottingham, UK) at 37 °C overnight, then centrifuged and transferred to the NGM plates^47^. L2 or L3 stage nematodes were transferred onto RNAi plates and left to grow and lay eggs for 4–5 days at 16 °C. These progenies were allowed to grow to L4 or young adults before molecular, cellular, or behavioral assays. For thrashing, at least 12 late L4 or young adult nematodes were placed in a drop of M9 buffer and allowed to recover for 30 s, then video recordings of the body bends for 30 s for each RNAi clone under the microscope were taken. For locomotion, at least 12 day 1 adults were randomly chosen from RNAi plates and transferred to a fresh plate seeded with OP50. After 5 min recovery, the number of body bends was counted per minute on each nematode. Each experiment was performed 3 times with independent biological replicates.

### Dopaminergic neuron impairment assay

For dopaminergic neuron observation, 60 nematodes at day 8 in each group were mounted onto agarose pads and paralyzed with 15 mM sodium azide. The micrographs for dopaminergic neurons were taken by a Zeiss confocal microscope LSM710 at 400× magnification. ZEN imaging software (Zeiss, Germany) was used to analyze the photos for each strain.

### Chemotaxis assay

*C. elegans* were cultivated at 16 °C until nematodes grew to L3 stage, then transferred to 23 °C until they grew to young adults. Animals were collected with M9 and washed 3 times. For each strain, animals were divided into naïve, trained, and control groups, each group contained at least 100 nematodes. For naïve group, animals were placed directly onto assay plates. For the trained group, animals were starved in M9 for 1 h, then transferred to NGM plates containing OP50, and 2 μL of 10% butanone was added to the lid for odor training for 1 h. For the control group, animals were starved in M9 for 1 h, then transferred to NGM plates containing OP50 without odor in the plates. After 1 h, these two plates of nematodes were washed with M9 and transferred to assay plates for observation. The number of worms was counted on both butanone and ethanol spots containing 1 μL of 1 M sodium azide after 1 h. Chemotaxis index (CI) and Learning index (LI) were calculated as Chemotaxis index (CI): [N_but_ − N_eth_] / [Total − N_origin_]; Learning index (LI): CI_but_ – CI_naive_^48^. Each experiment was performed 3 times with independent biological replicates.

### Western blot

*C. elegans* were cultivated at 16 °C until nematodes grew to L3 stage, then transferred to 23 °C until they grew to young adults. To extract H3K9me3 and ubiquitin proteins, *C. elegans* were collected with M9 buffer and transferred to RIPA buffer containing protease inhibitor. Animals were sonicated for 30 min (Bioruptor® Plus sonication device), then centrifuged at 12,000 g for 15 min to obtain the protein lysate. To extract the total α-syn, animals were collected with M9 buffer and resuspended in TBS-Urea-SDS buffer (1× TBS (pH 7.4), 5% SDS, 8 M Urea, 50 mM dithiothreitol (DTT), 1× protease inhibitor cocktail)^49^. Animals were sonicated for 30 min and centrifuged at 1500 g for 5 min to get rid of the debris. Supernatants were taken and further centrifuged at 14000 g for 20 min to obtain the protein lysate, this lysate contains soluble and insoluble α-syn in *C. elegans*. To extract the monomer and polymer forms of α-syn, animals were collected with M9 buffer and transferred to RAB buffer containing protease inhibitor. Animals were ground by pellet pestles, and then centrifuged at 15,000 g for 30 min to obtain protein lysate, this lysate mainly contains soluble α-syn in *C. elegans*.

Lysate supernatants were taken, and protein concentrations were measured by using the Pierce^TM^ BCA protein assay kit (Thermo Fisher Scientific). Twenty micrograms of proteins were loaded onto 12% SDS/PAGE or native/PAGE gels and blotted onto PVDF membranes (Bio-Rad). The primary antibody was incubated overnight at 4 °C, then changed to the secondary antibody and incubated for 1 h at room temperature. Antibody binding was visualized by using ECL Western Blotting Substrate (Bio-Rad). Antibodies used were anti-α-synuclein (LB509) (1:1000, Abcam, ab27766), anti-α-synuclein (1:1000, Thermo Fisher, PA5-85343), anti-β-actin (C4) (1:2000, Santa cruz, sc-47778), anti-H3K9me3 (1:2000, Abcam, ab8898), anti-Histone H3 (1:2000, Abcam, ab1791), anti-ubiquitin (1:2000, Proteintech, 10201-2-AP), Rabbit Anti-Mouse IgG H&L (HRP) (1:2000, Abcam, ab97046), Goat anti-rabbit IgG H&L (HRP) (1:2000, Proteintech, SA00001-2).

### Lysosome staining

*C. elegans* were cultivated at 16 °C until animals grew to L3 stage, then transferred to 23 °C until they grew to young adults. Animals were washed twice by M9 and soaked in M9 buffer containing 25 μM of lysotracker red and the same concentration of lysosensor blue (Invitrogen). Staining was carried out for 1 h at room temperature in dark. *C. elegans* were transferred to fresh NGM plates seeded with OP50 and allowed to recover for another 1 h^40^. Animals were picked up and paralyzed by 15 mM sodium azide and images were taken by a Zeiss confocal microscope LSM710 at 630× magnification. The fluorescence intensity of the lysosome was analyzed by using Image J software. At least 30 nematodes were scored for each strain.

### qRT-PCR

For gene expression, *C. elegans* were cultivated at 16 °C until nematodes grew to L3 stage, then transferred to 23 °C until they grew to young adults. *C. elegans* were collected in M9. RNAiso Plus (Takara) was used to extract total RNA. For mRNA expression, High-Capacity cDNA Reverse Transcription Kit (Applied Biosystems™) was used to convert RNA to cDNA. The qRT-PCR was performed using Power SYBR Green PCR Master Mix (Applied Biosystems™) and ABI 7500 system. The relative expression levels of genes were carried out using 2^−ΔΔCT^ method and normalized to the expression of gene *cdc-42*.

For small RNAs expression, Mir-X miRNA First-Strand Synthesis (Takara) was used to convert small RNA to cDNA. The qRT-PCR was performed using TG Green^TM^ Advantage qPCR Premix (Takara) and ABI 7500 system. The relative expression levels of piRNAs were carried out using 2^−ΔΔCT^ method and normalized to the expression of U6^5^. All the primers for genes and piRNAs expression are listed in Supplementary Data 8.

### RNA-Seq

*C. elegans* were cultivated at 16 °C until nematodes grew to L3 stage, then transferred to 23 °C until they grew to young adults. *C. elegans* were collected by M9, and total RNA was extracted by using RNAiso Plus (Takara). RNA samples were quantified by Bioanalyzer (Agilent 2100). RNA library was prepared following NEBNext® Ultra™ RNA Library Prep Kit for Illumina (New England Biolabs) and sequenced by Illumina HiSeq™ 2000 (FHS Genomics, Bioinformatics & Single Cell Core, University of Macau).

For quality control, adaptor sequences, low quality reads, and ambiguous reads were removed and then mapped to *C. elegans* genome (WBcel235) using HISAT2^50^. The genome sequence and gene annotation files were downloaded from the NCBI database. Samtools was used for sorting and Stringtie was used to estimate the abundances of transcripts and genes^51,52^. edgeR was used to perform differential expression analysis with thresholds as |log_2_(fold change) | >2, *P* value < 0.01 and FDR < 0.05^53^. Gene set enrichment analysis was performed in DAVID bioinformatics resources^54^. We refer to heat-shock protein genes as protein folding or protein degradation genes during function analysis. The protein products of these genes have dual related functions in both protein folding and protein degradation^55–57^.

For the sequencing data on α-syn(A53T) and Aβ;α-syn(A53T) strains, data were downloaded from a dataset with the accession number PRJNA622398 in NCBI SRA^19^. The analysis workflow was the same with our other samples in RNA sequencing with the thresholds adjusted to |log_2_(fold change)| > 1.5, *P* value < 0.01, and FDR < 0.05.

### Small RNA-Seq

*C. elegans* were cultivated at 16 °C until nematodes grew to L3 stage, then transferred to 23 °C until they grew to young adults. *C. elegans* were collected by M9, and total RNA was extracted by using RNAiso Plus (Takara). RNA samples were quantified by Bioanalyzer (Agilent 2100). A small RNA library was prepared following NEBNext® Multiplex Small RNA Library Prep Set for Illumina (New England Biolabs), size selection of the cDNA library was performed by 6% TBE PAGE gel (140 bp for miRNA, 150 bp for piRNA) (Thermo Fisher Scientific), and sequenced by Illumina HiSeq™ 2000. In order to keep all samples consistent, we collected wild type, α-syn(A53T)^Tg^, α-syn(A53T)^Tg^;*tofu-1(lf)*, Aβ^Tg^;α-syn(A53T)^Tg^ and Aβ^Tg^;α-syn(A53T)^Tg^;*tofu-1(lf)* strains, and sequenced all samples in one throughput submission to minimize technical variations or potential batch effects (FHS Genomics, Bioinformatics & Single Cell Core, University of Macau).

For quality control, adaptor sequences, low quality reads, and ambiguous reads were removed to obtain clean reads, which were then mapped to the *C. elegans* genome (WBcel235) using Bowtie (-a -v 0 -m 10)^58^. Samtools was used for sorting and the piRNA annotation files were downloaded from NCBI (WBcel235)^51^. featureCounts was used to measure the abundance of piRNA. edgeR was used to identify significant differentially expressed piRNA using threshold as *P* value < 0.01, FDR < 0.05, and |log_2_ (fold change)| > 1.5^53^. piRNA targets were predicted by using piRTarBase^31^. The enrichment analysis of piRNA targets was performed in DAVID bioinformatics resources^54^.

For 22G-RNAs analysis, potential 22G-RNAs were defined as 21-23 nt reads starting with G and using featureCounts with parameter (-s 2) to measure 22G-RNA antisense counts on the basis of their sequence being antisense to protein coding genes, transposons, simple repeats or piRNAs. edgeR was used to identify the differentially expressed 22G-RNAs coverage between different groups using threshold as *P* value < 0.01, FDR < 0.05 and |log_2_ (fold change)| > 1.5^53^.

### ChIP-Seq

*C. elegans* were cultivated at 16 °C until nematodes grew to L3 stage, then transferred to 23 °C until they grew to young adults. *C. elegans* were collected and washed by M9 buffer three times. The supernatant was discarded to obtain a tightly packed pellet and the nematodes were placed into liquid nitrogen immediately. Frozen nematodes were ground using a pestle and suspended in 1% fresh formaldehyde in PBS solution to cross-link proteins to DNA, and then incubated at room temperature on a shaker for 10 min. The crosslinking reaction was quenched by adding 2 M glycine and incubated for 5 min. *C. elegans* was washed three times with cold PBS (contain 1× proteinase inhibitor cocktail) and kept in −80 °C^59^. The *C. elegans* pellet was resuspended in nuclei preparation buffer (Imprint® Chromatin Immunoprecipitation Kit) and sonicated for 18 min with 30 s ON and 30 s OFF (Bioruptor® Plus sonication device) to get the 150–500 bp of DNA fragments. Chip reactions were carried out using 2–5 μg chromatin DNA, 5 μL of diluted supernatant was removed as “input DNA”, then 2 μg of H3K9me3 antibody was added (Abcam, ab8898). After 60–90 min, samples were reverse crosslinked to obtain the genomic DNA.

For ChIP-Seq, genomic DNA was purified. The DNA library was prepared following the protocol of NEBNext® Ultra™ II DNA Library Prep Kit for Illumina® (New England Biolabs), and sequencing was performed by Illumina HiSeq™ 2000 (FHS Genomics, Bioinformatics & Single Cell Core, University of Macau).

For quality control, adaptor sequences, low quality reads, and ambiguous reads were removed to obtain clean reads, which were then mapped to the *C. elegans* genome (WBcel235) using Bowtie2^60^. Samtools were used for sorting and peaks were identified by using MACS2^51,61^. edgeR was used to identify significant differentially modified peaks between two groups using threshold as *P* value < 0.05 and |log_2_ (fold change)| > 2^53^. Chipseeker was used to annotate the differentially modified peaks^62^. The heatmaps, read distribution of differentially modified peaks were drawn by using deepTools^63^. The enrichment analysis of differentially H3K9me3 modified genes were performed in DAVID bioinformatics resources^54^.

### ChIP-qPCR

For ChIP-qPCR, genomic DNA was purified and then analyzed by using Power SYBR Green PCR Master Mix (Applied Biosystems™) and ABI 7500 system. The calculation of the % Input for each ChIP fraction was 2^(−ΔCt [normalized ChIP])^, where ΔCt [normalized ChIP] = (Ct [ChIP] - (Ct [Input] - Log_2_(Input Dilution Factor))). Primers used are listed in Supplementary Data 8.

### Bioinformatic analysis of PD patient samples

Non-coding RNA profiling dataset (GSE97285) including 7 early-premotor PD samples, 7 late-motor stage PD samples, and 14 control samples were downloaded and analyzed to obtain the differentially expressed piRNAs in PD patients^43^. Samples were obtained from the amygdala. The raw data were downloaded from SRA database using prefetch and decompressed by fastq-dump. The human genome sequence file (GRCh38) was downloaded from NCBI database. The piRNA annotation files were downloaded from piRBase^64^, respectively. Read alignment was performed by Bowtie (-a -v 0 -m 10)^55^. Samtools was used for sorting and featureCounts was used to measure the piRNA abundance^51,65^. edgeR was used to do differential analysis using threshold as *P* value < 0.01 and |log_2_ (fold change)| > 1^53^.

### Statistical analysis

*P* values were calculated by one-way ANOVA, followed by a Tukey Post-Hoc test to compare each group. Two-tailed student’s *t-*test was used only in two group comparisons. The difference was considered significant when *P* < 0.05. Data are shown as mean ± SEM. Kaplan-Meier was carried out and *P* values were calculated using log-rank test in lifespan analysis. Statistical analyses were carried out using IBM SPSS statistic 20. Figures were drawn by OriginPro 8 or GraphPad Prism 8, and further imported to Adobe Illustrator CC 2018.

### Reporting summary

Further information on research design is available in the Nature Portfolio Reporting Summary linked to this article.

### Supplementary information

Supplementary Information

Description of Additional Supplementary Files

Supplementary Data 1

Supplementary Data 2

Supplementary Data 3

Supplementary Data 4

Supplementary Data 5

Supplementary Data 6

Supplemenatry Data 7

Supplementary Data 8

Reporting Summary

### Source data

Source Data

## Data Availability

All relevant data generated or analyzed during this study are included in this manuscript and/or its supplementary information. The data underlying Figures and Supplementary Figures are provided as Source data. The Raw RNA, and small RNA and ChIP-Seq data generated in this study have been deposited to NCBI SRA database; the accession numbers are PRJNA732252 for RNA-Seq, PRJNA622394 for small RNA-Seq, PRJNA983087 for t*ofu-1* mutant small RNA-Seq, and PRJNA763505 for ChIP-Seq, respectively. Source data are provided with this paper.

## References

[1] Vagin VV (2006). A distinct small RNA pathway silences selfish genetic elements in the germline. Science.

[2] Siomi MC, Sato K, Pezic D, Aravin AA (2011). PIWI-interacting small RNAs: the vanguard of genome defence. Nat. Rev. Mol. Cell Biol..

[3] Iwasaki YW, Siomi MC, Siomi H (2015). PIWI-interacting RNA: its biogenesis and functions. Annu. Rev. Biochem..

[4] Izumi N, Tomari Y (2014). Diversity of the piRNA pathway for nonself silencing: worm-specific piRNA biogenesis factors. Genes Dev..

[5] Goh WS (2014). A genome-wide RNAi screen identifies factors required for distinct stages of C. elegans piRNA biogenesis. Genes Dev..

[6] Ozata DM, Gainetdinov I, Zoch A, O’Carroll D, Zamore PD (2019). PIWI-interacting RNAs: small RNAs with big functions. Nat. Rev. Genet..

[7] Sienski G, Donertas D, Brennecke J (2012). Transcriptional silencing of transposons by Piwi and maelstrom and its impact on chromatin state and gene expression. Cell.

[8] Luteijn MJ, Ketting RF (2013). PIWI-interacting RNAs: from generation to transgenerational epigenetics. Nat. Rev. Genet..

[9] Huang X, Wong G (2021). An old weapon with a new function: PIWI-interacting RNAs in neurodegenerative diseases. Transl. Neurodegener..

[10] Rajasethupathy P (2012). A role for neuronal piRNAs in the epigenetic control of memory-related synaptic plasticity. Cell.

[11] Lee EJ (2011). Identification of piRNAs in the central nervous system. Rna.

[12] Kim KW (2018). A neuronal piRNA pathway inhibits axon regeneration in C. elegans. Neuron.

[13] Moore RS, Kaletsky R, Murphy CT (2019). Piwi/PRG-1 argonaute and TGF-beta mediate transgenerational learned pathogenic avoidance. Cell.

[14] Kaletsky R (2020). C. elegans interprets bacterial non-coding RNAs to learn pathogenic avoidance. Nature.

[15] Sun W, Samimi H, Gamez M, Zare H, Frost B (2018). Pathogenic tau-induced piRNA depletion promotes neuronal death through transposable element dysregulation in neurodegenerative tauopathies. Nat. Neurosci..

[16] Soto C, Pritzkow S (2018). Protein misfolding, aggregation, and conformational strains in neurodegenerative diseases. Nat. Neurosci..

[17] Outeiro TF (2019). Dementia with Lewy bodies: an update and outlook. Mol. Neurodegener..

[18] Lakso M (2003). Dopaminergic neuronal loss and motor deficits in Caenorhabditis elegans overexpressing human alpha-synuclein. J. Neurochem..

[19] Huang X (2021). Human amyloid beta and alpha-synuclein co-expression in neurons impair behavior and recapitulate features for Lewy body dementia in Caenorhabditis elegans. Biochim. et. Biophys. acta Mol. Basis Dis..

[20] Shen L, Wang C, Chen L, Wong G (2021). Dysregulation of MicroRNAs and PIWI-interacting RNAs in a caenorhabditis elegans parkinson’s disease model overexpressing human α-synuclein and influence of tdp-1. Front Neurosci..

[21] Schulze M (2018). Sporadic Parkinson’s disease derived neuronal cells show disease-specific mRNA and small RNA signatures with abundant deregulation of piRNAs. Acta Neuropathol. Commun..

[22] Qiu W (2017). Transcriptome-wide piRNA profiling in human brains of Alzheimer’s disease. Neurobiol. Aging.

[23] Roy J, Sarkar A, Parida S, Ghosh Z, Mallick B (2017). Small RNA sequencing revealed dysregulated piRNAs in Alzheimer’s disease and their probable role in pathogenesis. Mol. Biosyst..

[24] Winston WM, Molodowitch C, Hunter CP (2002). Systemic RNAi in C. elegans requires the putative transmembrane protein SID-1. Science.

[25] Kumsta C, Hansen M (2012). C. elegans rrf-1 mutations maintain RNAi efficiency in the soma in addition to the germline. PloS ONE.

[26] Kalia LV, Lang AE (2015). Parkinson’s disease. Lancet (Lond., Engl.).

[27] Arnaoutoglou NA, O’Brien JT, Underwood BR (2019). Dementia with Lewy bodies - from scientific knowledge to clinical insights. Nat. Rev. Neurol..

[28] Maulik M, Mitra S, Bult-Ito A, Taylor BE, Vayndorf EM (2017). Behavioral phenotyping and pathological indicators of Parkinson’s disease in C. elegans models. Front. Genet..

[29] Bagijn MP (2012). Function, targets, and evolution of Caenorhabditis elegans piRNAs. Science.

[30] Batista PJ (2008). PRG-1 and 21U-RNAs interact to form the piRNA complex required for fertility in C. elegans. Mol. Cell.

[31] Wu WS (2019). piRTarBase: a database of piRNA targeting sites and their roles in gene regulation. Nucleic Acids Res..

[32] Lu Z, Hunter T (2009). Degradation of activated protein kinases by ubiquitination. Annu. Rev. Biochem..

[33] Dehay B (2012). Lysosomal dysfunction in Parkinson disease: ATP13A2 gets into the groove. Autophagy.

[34] Wallings RL, Humble SW, Ward ME, Wade-Martins R (2019). Lysosomal dysfunction at the centre of Parkinson’s disease and frontotemporal dementia/amyotrophic lateral sclerosis. Trends Neurosci..

[35] Ihara Y, Morishima-Kawashima M, Nixon R (2012). The ubiquitin-proteasome system and the autophagic-lysosomal system in Alzheimer disease. Cold Spring Harb. Perspect. Med..

[36] Bonam SR, Wang F, Muller S (2019). Lysosomes as a therapeutic target. Nat. Rev. Drug Discov..

[37] Mazzulli JR, Zunke F, Isacson O, Studer L, Krainc D (2016). alpha-Synuclein-induced lysosomal dysfunction occurs through disruptions in protein trafficking in human midbrain synucleinopathy models. Proc. Natl Acad. Sci. USA.

[38] Guerreiro R (2018). Investigating the genetic architecture of dementia with Lewy bodies: a two-stage genome-wide association study. Lancet Neurol..

[39] Blauwendraat C (2020). Genetic modifiers of risk and age at onset in GBA associated Parkinson’s disease and Lewy body dementia. Brain: J. Neurol..

[40] Sun Y (2020). Lysosome activity is modulated by multiple longevity pathways and is important for lifespan extension in C. elegans. eLife.

[41] Vidoni C, Follo C, Savino M, Melone MA, Isidoro C (2016). The Role of Cathepsin D in the Pathogenesis of Human Neurodegenerative Disorders. Med. Res. Rev..

[42] Settembre C (2011). TFEB links autophagy to lysosomal biogenesis. Science.

[43] Pantano L (2016). Specific small-RNA signatures in the amygdala at premotor and motor stages of Parkinson’s disease revealed by deep sequencing analysis. Bioinforma. (Oxf., Engl.).

[44] Sidransky E (2009). Multicenter analysis of glucocerebrosidase mutations in Parkinson’s disease. N. Engl. J. Med..

[45] Guo C (2018). Tau Activates Transposable Elements in Alzheimer’s Disease. Cell Rep..

[46] Nussbaum-Krammer C. I., Neto M. F., Brielmann R. M., Pedersen J. S., Morimoto R. I. Investigating the spreading and toxicity of prion-like proteins using the metazoan model organism C. elegans. *J. Vis. Exp.: JoVE*, 52321 (2015). 10.3791/52321 PMC4354510 25591151

[47] Zhang H, Abraham N, Khan LA, Gobel V (2015). RNAi-based biosynthetic pathway screens to identify in vivo functions of non-nucleic acid-based metabolites such as lipids. Nat. Protoc..

[48] Wang C, Saar V, Leung KL, Chen L, Wong G (2018). Human amyloid beta peptide and tau co-expression impairs behavior and causes specific gene expression changes in Caenorhabditis elegans. Neurobiol. Dis..

[49] Bandopadhyay R. Sequential Extraction of Soluble and Insoluble Alpha-Synuclein from Parkinsonian Brains. *J. Vis. Exp.: JoVE*, 53415 (2016). 10.3791/53415 PMC4781043 26780369

[50] Pertea M, Kim D, Pertea GM, Leek JT, Salzberg SL (2016). Transcript-level expression analysis of RNA-seq experiments with HISAT, StringTie and Ballgown. Nat. Protoc..

[51] Li H (2009). The sequence alignment/map format and SAMtools. Bioinforma. (Oxf., Engl.).

[52] Pertea M (2015). StringTie enables improved reconstruction of a transcriptome from RNA-seq reads. Nat. Biotechnol..

[53] Robinson MD, McCarthy DJ, Smyth GK (2010). edgeR: a Bioconductor package for differential expression analysis of digital gene expression data. Bioinforma. (Oxf., Engl.).

[54] Huang da W, Sherman BT, Lempicki RA (2009). Systematic and integrative analysis of large gene lists using DAVID bioinformatics resources. Nat. Protoc..

[55] Mathew A, Mathur SK, Morimoto RI (1998). Heat shock response and protein degradation: regulation of HSF2 by the ubiquitin-proteasome pathway. Mol. Cell. Biol..

[56] Leak RK (2014). Heat shock proteins in neurodegeneration disorders and aging. J. Cell Commun. Signal.

[57] Fernández-Fernández MR, Gragera M, Ochoa-Ibarrola L, Quintana-Gallardo L, Valpuesta JM (2017). Hsp70 – a master regulator in protein degradation. FEBS Lett..

[58] Langmead B, Trapnell C, Pop M, Salzberg SL (2009). Ultrafast and memory-efficient alignment of short DNA sequences to the human genome. Genome Biol..

[59] Yuan J (2020). Two conserved epigenetic regulators prevent healthy ageing. Nature.

[60] Langmead B, Salzberg SL (2012). Fast gapped-read alignment with Bowtie 2. Nat. Methods.

[61] Zhang Y (2008). Model-based analysis of ChIP-Seq (MACS). Genome Biol..

[62] Yu G, Wang LG, He QY (2015). ChIPseeker: an R/Bioconductor package for ChIP peak annotation, comparison and visualization. Bioinforma. (Oxf., Engl.).

[63] Ramirez F (2016). deepTools2: a next generation web server for deep-sequencing data analysis. Nucleic Acids Res..

[64] Wang J (2018). piRBase: a comprehensive database of piRNA sequences. Nucleic Acids Res..

[65] Liao Y, Smyth GK, Shi W (2014). featureCounts: an efficient general purpose program for assigning sequence reads to genomic features. Bioinforma. (Oxf., Engl.).

