# Supplementary material for: PIWI-interacting RNA expression regulates pathogenesis in a Caenorhabditis elegans model of Lewy body disease
Source: Nat Commun. 2023 Oct 2;14:6137. doi: 10.1038/s41467-023-41881-8 (PMC10545829; doi:10.1038/s41467-023-41881-8)
Supplement: Supplementary file 1 — Supplementary Information [file 41467_2023_41881_MOESM1_ESM.pdf]

Supplementary Information for

PIWI-interacting RNA expression regulates pathogenesis in a *Caenorhabditis elegans* model of Lewy body disease

Xiaobing Huang, Changliang Wang, Tianjiao Zhang, Rongzhen Li, Liang Chen, Ka Lai Leung, Merja Lakso, Qinghua Zhou, Hongjie Zhang, Garry Wong*

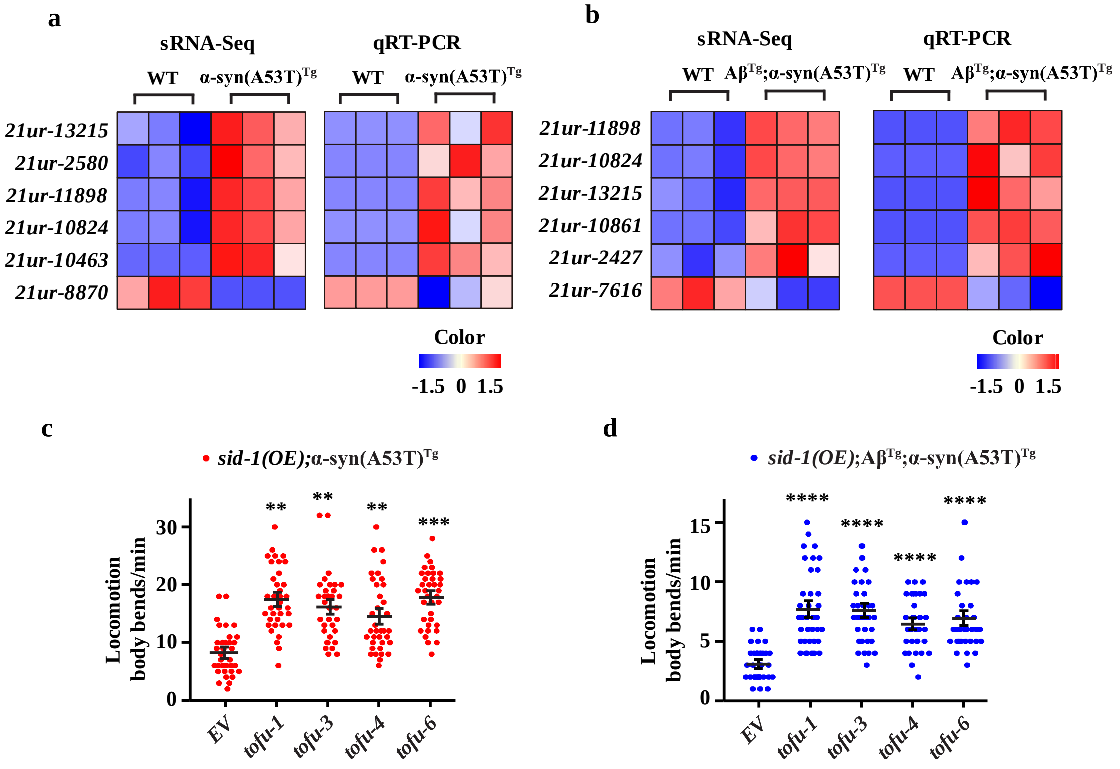

Supplementary Fig. 1 piRNA expression and RNAi screening in transgenic animals. a, b Small RNA-Seq (sRNA-Seq) and qRT-PCR to check piRNAs expression in WT *vs* α-syn(A53T)^{Tg} and WT *vs* Aβ^{Tg};α-syn(A53T)^{Tg}. Samples were collected for each strain for qRT-PCR in each group. Three biological replicates were performed. Data was shown as logCPM in small RNA-Seq, and relative expression change in qRT-PCR. The same piRNAs expression trend in qRT-PCR and small RNA-Seq. **c, d** Locomotion assay in transgenic strains under *tofu-1*, *tofu-3*, *tofu-4*, and *tofu-6* RNAi clones. At least 12 nematodes were calculated for each RNAi clone, 3 biological replications (mean ±SEM, one-way ANOVA). **** P<0.0001, *** P<0.001, ** P<0.01. Source data are provided as a Source Data file.

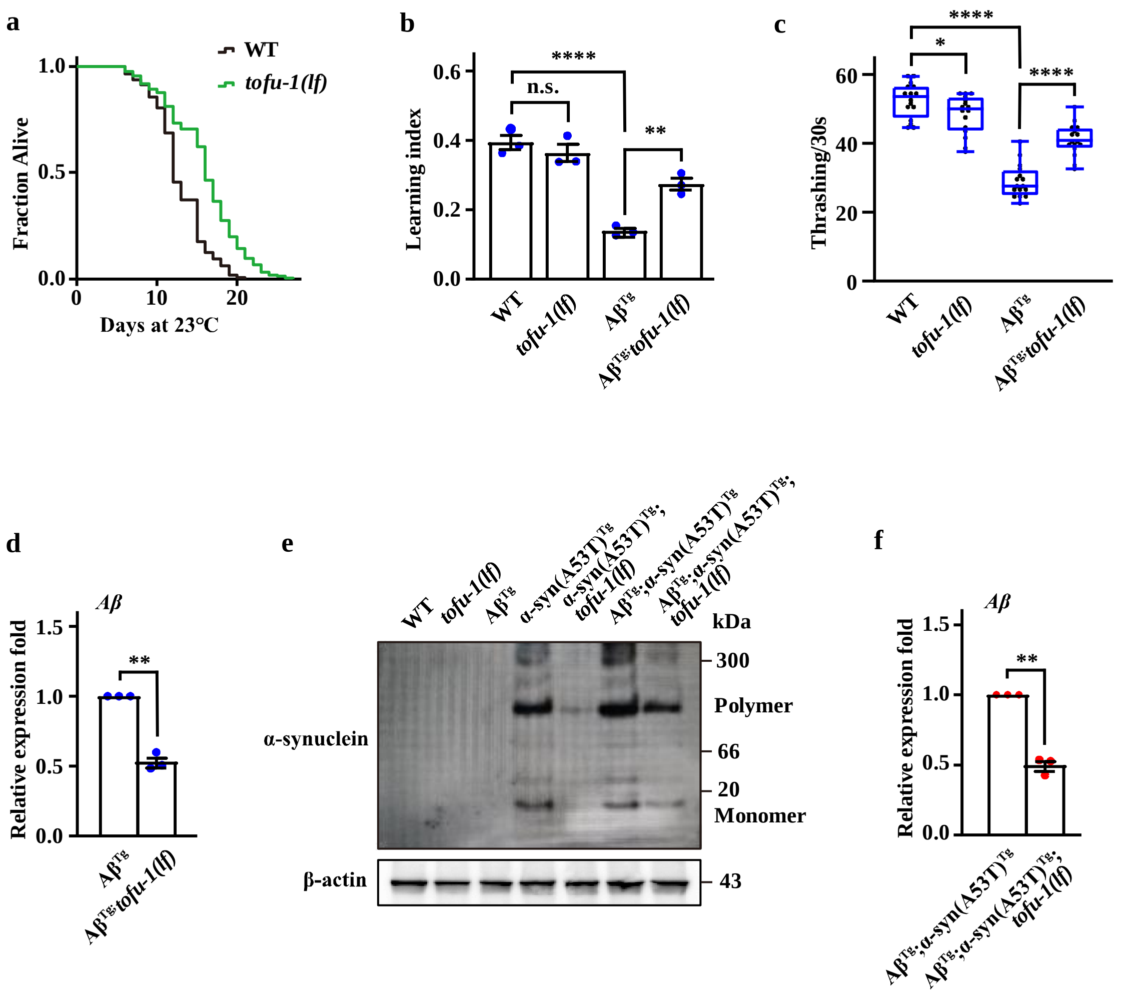

Supplementary Fig. 2 TOFU-1 deletion ameliorates behavioral impairments. a The survival curve of *tofu-1* mutant compared with wild type. Over 70 nematodes were analyzed in each group for each experiment with 3 biological replicates. The average lifespan was analyzed by Kaplan-Meier test, *P* value was calculated by the log-rank test. **b** Learning index in transgenic animals (mean ±SEM, n=3, one-way ANOVA). **c** Thrashing assay in transgenic animals. At least 12 nematodes were calculated for each RNAi clone, 3 biological replications (one-way ANOVA). The box plots display the median, upper and lower quartiles; the whiskers show 1.5× interquartile range (IQR). **d** and **f** Aβ gene expression in transgenic animals (mean ±SEM, n=3, two-tailed student's *t*-test). **e** Native gel to analysis of monomer and polymer form of α-syn expression in transgenic animals at young adult stage. **** $P<0.0001$, ** $P<0.01$, * $P<0.05$, n.s. not significant. Source data are provided as a Source Data file.

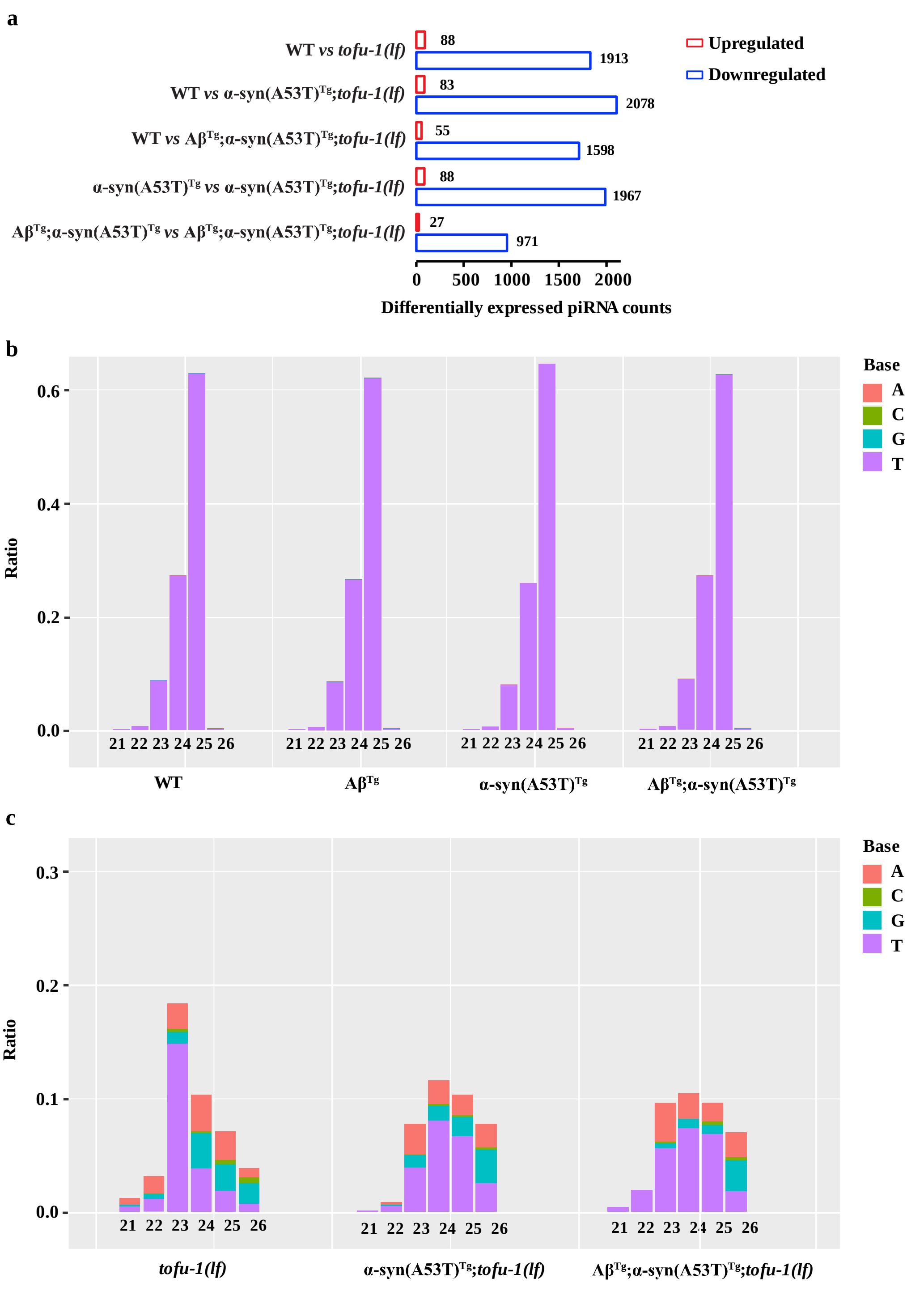

Supplementary Fig. 3 piRNA expression after *tofu-1* deletion in transgenic animals. a Numbers of differentially expressed piRNAs (DEPs) in different transgenic strains. Red column indicated the upregulated numbers of DEPs and blue column indicated the downregulated numbers of DEPs in each group. Data for differentially expressed piRNAs are provided in Supplementary Data 1. **b, c** Length distribution of piRNAs in transgenic strains.

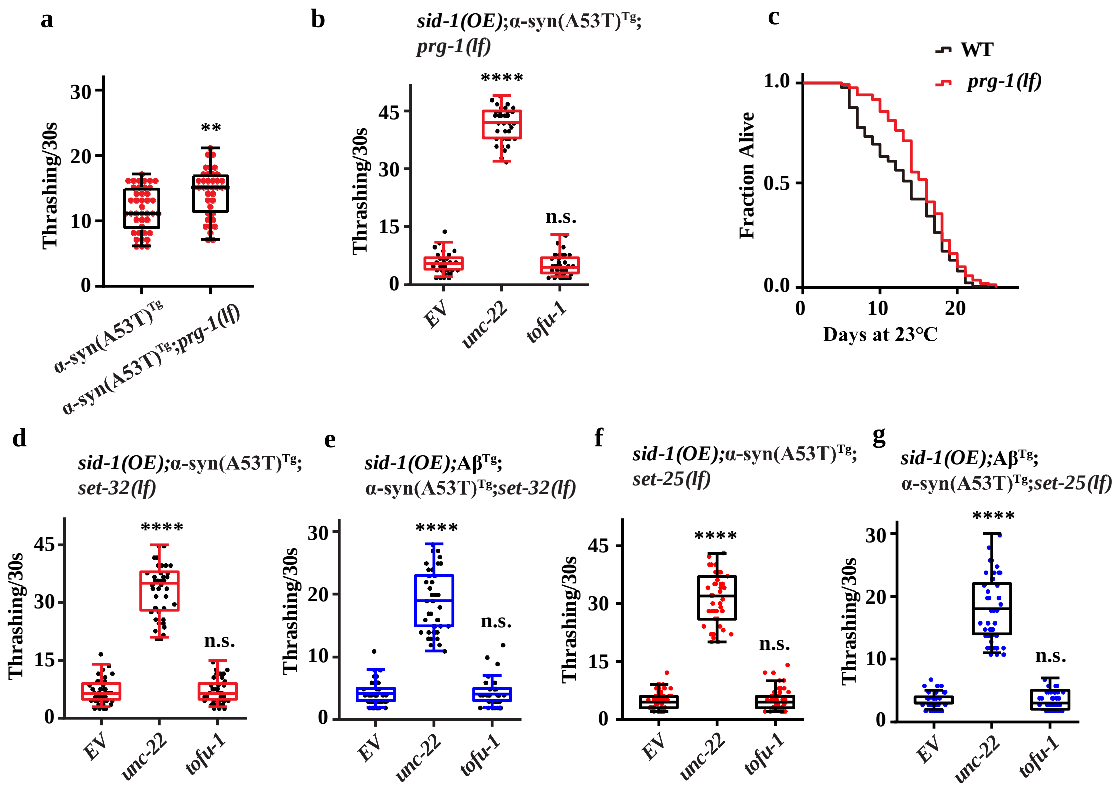

Supplementary Fig. 4 Thrashing and lifespan in transgenic animals. a Thrashing assay in α-syn(A53T) strain before and after *prg-1* gene deletion in *C. elegans*. **b** and **d-g** Thrashing assay in transgenic animals under RNAi. *EV* was empty vector (negative control), and *unc-22* was the positive control for RNAi. For **a, b, d-g** At least 12 nematodes were calculated for each group, 3 biological replications (mean ±SEM, one-way ANOVA). The box plots display the median, upper and lower quartiles; the whiskers show 1.5× interquartile range (IQR). **c** The survival curve of *prg-1* mutant compared with wild type. The average lifespan was analyzed by Kaplan-Meier test, *P* value was calculated by the log-rank test. **** $P<0.0001$, ** $P<0.01$, * $P<0.05$, n.s. not significant. Source data are provided as a Source Data file.

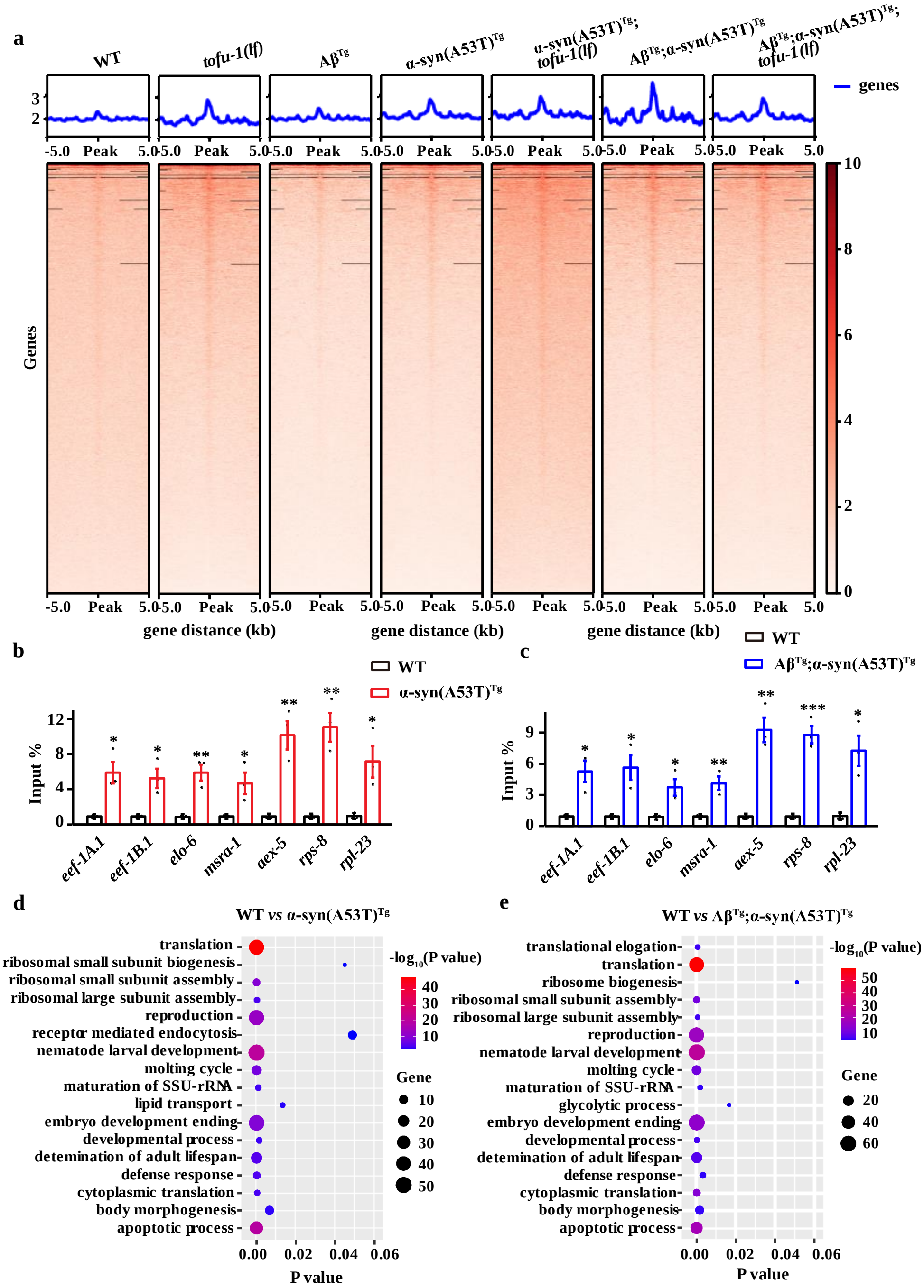

Supplementary Fig. 5 ChIP-Seq in transgenic animals. **a** Heatmap of H3K9me3 ChIP signals around 5kb upstream and downstream of regions from peaks that were differentially enriched in *C. elegans*. The peaks were ranked in descending order of H3K9me3 intensity within each cluster. Data was shown in Supplementary Data 3. **b, c** ChIP-qPCR of H3K9me3 in transgenic strains (mean ± SEM, n=3, two-tailed student's *t*-test). *P<0.05, **P<0.01, *** P<0.001. **d, e** GO analysis in differentially modified genes in ChIP-Seq. GO terms are shown on the left side of the chart. All of these terms are upregulated. The size of circles denotes the number of genes in the terms that are regulated. -$\log_{10}$(P value) is indicated by the color of the circle and the P value is indicated by the location on the horizontal axis.

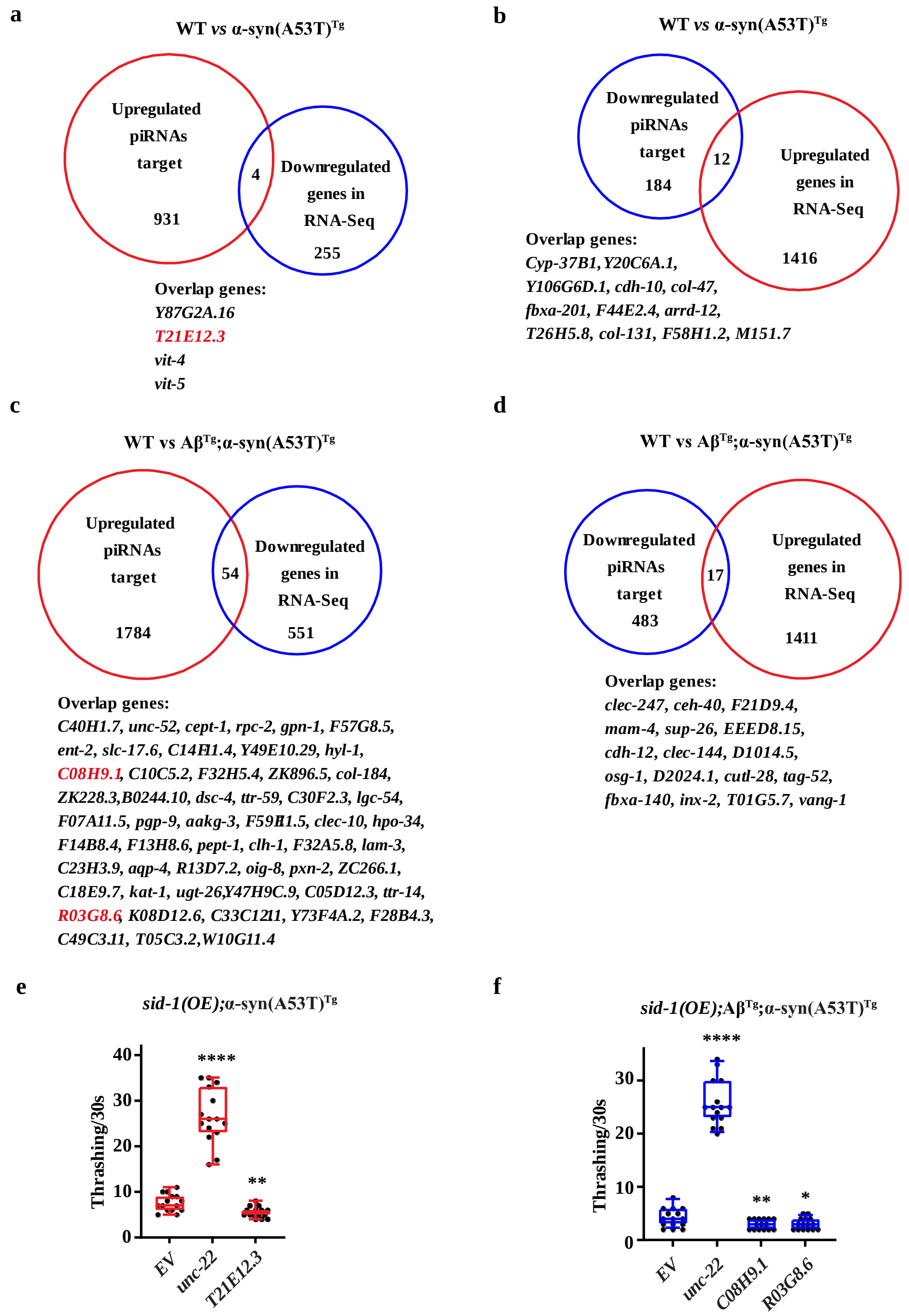

Supplementary Fig. 6 piRNA target gene analysis. a-d Venn diagrams of overlapping regulated piRNA target genes and RNA-Seq. (**a**) and **(c)** An overlapped 4 and 54 genes were found in upregulated piRNAs target prediction and downregulated DEGs in RNA-Seq in WT *vs* α-syn(A53T)^{Tg} and WT *vs* Aβ^{Tg};α-syn(A53T)^{Tg}. (**b**) and (**d**) An overlapped 12 and 17 genes were found in downregulated piRNAs target genes and upregulated DEGs in RNA-Seq in WT *vs* α-syn(A53T)^{Tg} and WT *vs* Aβ^{Tg};α-syn(A53T)^{Tg}. Data for RNA-Seq were downloaded from dataset with the accession number PRJNA622398 in NCBI SRA. **e, f** Thrashing assay in transgenic animals under RNAi At least 12 nematodes were calculated for each RNAi clone, 3 biological replications (mean ±SEM, one-way ANOVA)., **** $P<0.0001$, **$P<0.01$, *$P<0.05$.

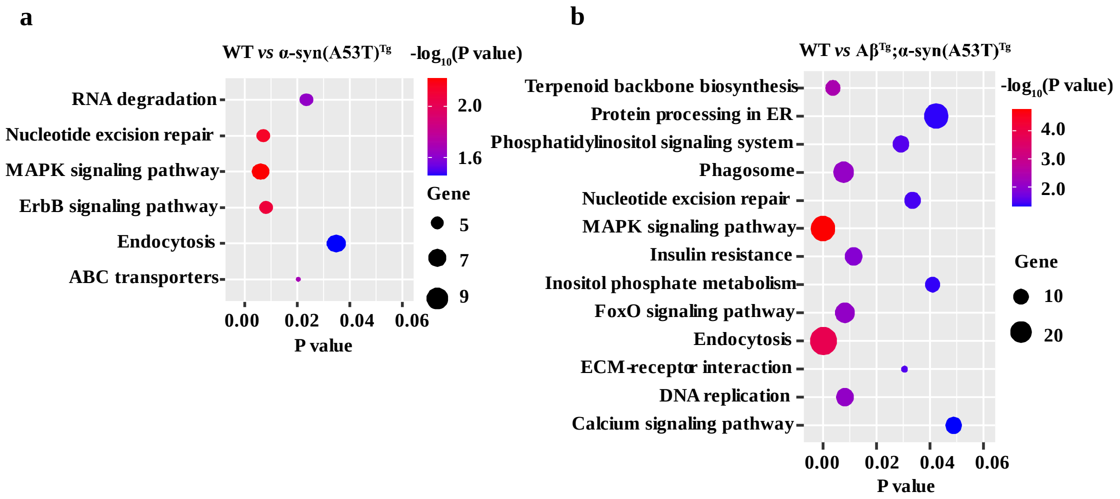

Supplementary Fig. 7 Enrichment analysis for upregulated piRNA targets in transgenic animals. a, b Enriched KEGG analysis in WT *vs* α-syn(A53T)^{Tg} and WT *vs* Aβ^{Tg};α-syn(A53T)^{Tg} for upregulated piRNA target genes. Pathways are shown on the left side of the chart. The size of balls denotes the numbers of genes in pathways that are regulated. -log$_{10}$ (P value) is indicated by the color of the circle and the P value is indicated by the location on the horizontal axis.

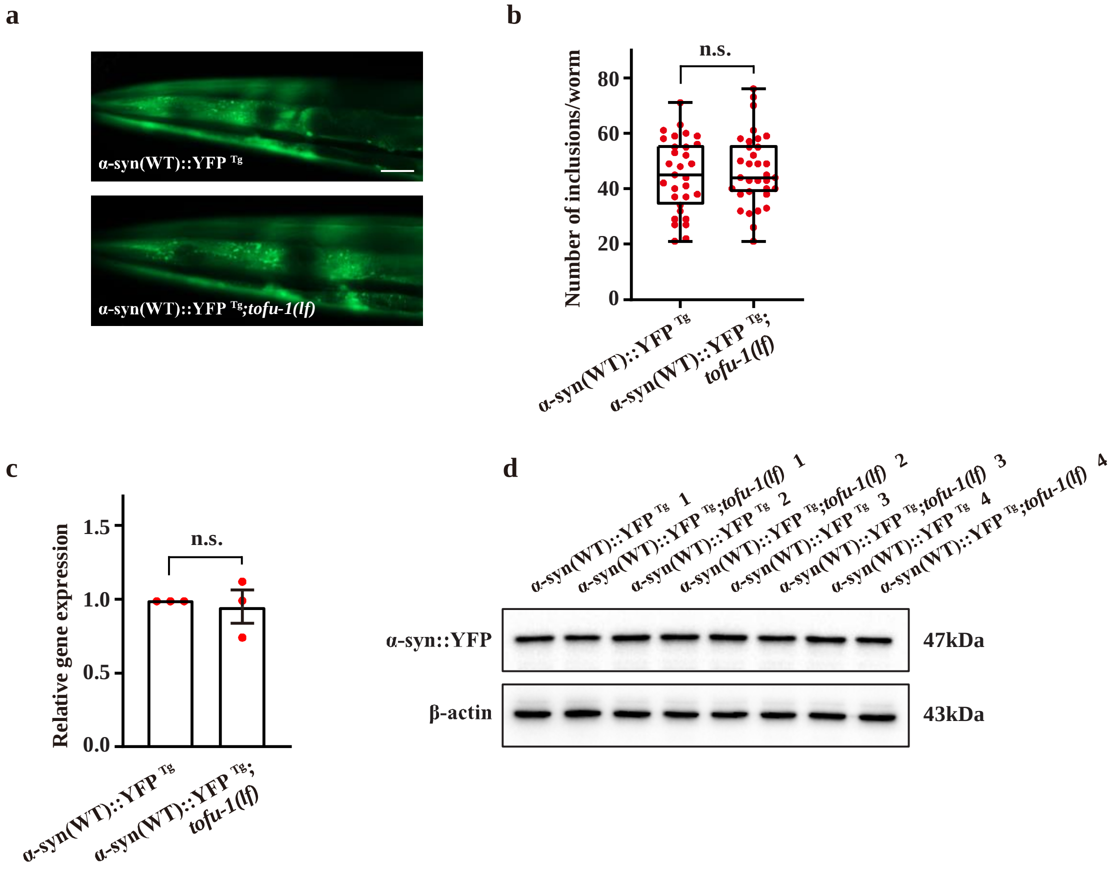

Supplementary Fig. 8 α-syn expression in α-syn(WT)::YFP ^{Tg} transgenic strain. a Imaging of α-syn inclusion in α-syn(WT)::YFP ^{Tg} and α-syn(WT)::YFP ^{Tg}; *tofu-1(lf)* strains in young adult stage. Over 30 animals were calculated for each strain. Scale bar = 20 μM. **b** Quantitation of inclusions. The box plots display the median, upper and lower quartiles; the whiskers show 1.5× interquartile range (IQR). **c** α-syn gene expression in transgenic animals (mean ± SEM, n=3, two-tailed student's *t*-test). **d** Western blot to check α-syn expression with 4 biological replicates in transgenic animals. n.s. not significant. Source data are provided as a Source Data file.
