# Supplementary material for: PIWI-interacting RNA expression regulates pathogenesis in a Caenorhabditis elegans model of Lewy body disease
Source: Nat Commun. 2023 Oct 2;14:6137. doi: 10.1038/s41467-023-41881-8 (PMC10545829; doi:10.1038/s41467-023-41881-8)
Supplement: Supplementary file 2 — Description of Additional Supplementary Files [file 41467_2023_41881_MOESM2_ESM.pdf]

Description of Additional Supplementary Files

Supplementary Data 1. Differentially expressed piRNAs in small RNA-Seq

Supplementary Data 2. RNAi screening gene list

Supplementary Data 3. Differentially modified genes in ChIP-Seq

Supplementary Data 4. Differentially expressed 22G-RNAs in small RNA-Seq

Supplementary Data 5. Differentially expressed genes in RNA-Seq

Supplementary Data 6. Differentially expressed piRNAs in PD patients

Supplementary Data 7. Strain list

Supplementary Data 8. Primer used in this project
