# Supplementary material for: PIWI-interacting RNA expression regulates pathogenesis in a Caenorhabditis elegans model of Lewy body disease
Source: Nat Commun. 2023 Oct 2;14:6137. doi: 10.1038/s41467-023-41881-8 (PMC10545829; doi:10.1038/s41467-023-41881-8)
Supplement: Supplementary file 11 — Reporting Summary [file 41467_2023_41881_MOESM11_ESM.pdf]

nature portfolio

Corresponding author(s): Garry Wong

Last updated by author(s): 22 December 2022

Reporting Summary

Nature Portfolio wishes to improve the reproducibility of the work that we publish. This form provides structure for consistency and transparency in reporting. For further information on Nature Portfolio policies, see our Editorial Policies and the Editorial Policy Checklist.

Statistics

For all statistical analyses, confirm that the following items are present in the figure legend, table legend, main text, or Methods section.

n/a	Confirmed	
☐	☒	The exact sample size (n) for each experimental group/condition, given as a discrete number and unit of measurement
☐	☒	A statement on whether measurements were taken from distinct samples or whether the same sample was measured repeatedly
☐	☒	The statistical test(s) used AND whether they are one- or two-sided<br>*Only common tests should be described solely by name; describe more complex techniques in the Methods section.*
☐	☒	A description of all covariates tested
☐	☒	A description of any assumptions or corrections, such as tests of normality and adjustment for multiple comparisons
☐	☒	A full description of the statistical parameters including central tendency (e.g. means) or other basic estimates (e.g. regression coefficient) AND variation (e.g. standard deviation) or associated estimates of uncertainty (e.g. confidence intervals)
☐	☒	For null hypothesis testing, the test statistic (e.g. F, t, r) with confidence intervals, effect sizes, degrees of freedom and P value noted<br>*Give P values as exact values whenever suitable.*
☒	☐	For Bayesian analysis, information on the choice of priors and Markov chain Monte Carlo settings
☐	☒	For hierarchical and complex designs, identification of the appropriate level for tests and full reporting of outcomes
☒	☐	Estimates of effect sizes (e.g. Cohen's d, Pearson's r), indicating how they were calculated

Our web collection on statistics for biologists contains articles on many of the points above.

Software and code

Policy information about availability of computer code

Data collection	Image J (V 1.8.0), ZEN imaging software (LSM710), ABI 7500, Agilent Bioanalyzer 2100 system, Illumina HiSeq™ 2000.
Data analysis	Excel (2020), SPSS 20, Origin 2018, Adobe Illustrator CC 2018, R 4.1.2, R studio, DAVID Bioinformatics Resources.

For manuscripts utilizing custom algorithms or software that are central to the research but not yet described in published literature, software must be made available to editors and reviewers. We strongly encourage code deposition in a community repository (e.g. GitHub). See the Nature Portfolio guidelines for submitting code & software for further information.

Data

Policy information about availability of data

All manuscripts must include a data availability statement. This statement should provide the following information, where applicable:

- Accession codes, unique identifiers, or web links for publicly available datasets
- A description of any restrictions on data availability
- For clinical datasets or third party data, please ensure that the statement adheres to our policy

PRJNA732252 for RNA-Seq, PRJNA622394 for small RNA-Seq and PRJNA763505 for Chip-seq

Human research participants

Policy information about studies involving human research participants and Sex and Gender in Research.

Reporting on sex and gender	Both genders reported
Population characteristics	Population characteristics reported
Recruitment	Data was publically available, recruitment data is associated with original study
Ethics oversight	Data was publically available, ethics oversight covered by original study

Note that full information on the approval of the study protocol must also be provided in the manuscript.

Field-specific reporting

Please select the one below that is the best fit for your research. If you are not sure, read the appropriate sections before making your selection.

☒ Life sciences ☐ Behavioural & social sciences ☐ Ecological, evolutionary & environmental sciences

For a reference copy of the document with all sections, see nature.com/documents/nr-reporting-summary-flat.pdf

Life sciences study design

All studies must disclose on these points even when the disclosure is negative.

Sample size	No sample size calculations were performed
Data exclusions	No data excluded
Replication	Replications were performed and successful
Randomization	Samples were randomly allocated into experimental groups
Blinding	Investigators were not blinded

Reporting for specific materials, systems and methods

We require information from authors about some types of materials, experimental systems and methods used in many studies. Here, indicate whether each material, system or method listed is relevant to your study. If you are not sure if a list item applies to your research, read the appropriate section before selecting a response.

Materials & experimental systems

n/a	Involved in the study	
☐	☒	Antibodies
☒	☐	Eukaryotic cell lines
☒	☐	Palaeontology and archaeology
☐	☒	Animals and other organisms
☒	☐	Clinical data
☒	☐	Dual use research of concern

Methods

n/a	Involved in the study	
☐	☒	ChIP-seq
☒	☐	Flow cytometry
☒	☐	MRI-based neuroimaging

Antibodies

Antibodies used	anti-α-synuclein (LB509) (1:1000, Abcam, ab27766), anti-β-actin (C4) (1:2000, Santa cruz , sc-47778), anti-H3K9me3 (1:2000, Abcam, ab8898), anti-Histone H3 (1:2000, Abcam, ab1791), anti-ubiquitin (1:2000, Proteintech, 10201-2-AP), Rabbit Anti-Mouse IgG H&L (HRP) (1:2000, Abcam, ab97046), Goat anti-rabbit IgG H&L (HRP) (1:2000, Proteintech, SA00001-2)
Validation	Manufacturer's website

Animals and other research organisms

Policy information about studies involving animals; ARRIVE guidelines recommended for reporting animal research, and Sex and Gender in Research

Laboratory animals	Caenorhabditis elegans, hermaphrodite
Wild animals	No wild animals were used in this study.
Reporting on sex	For Caenorhabditis elegans, , we used hermaphrodite and male.
Field-collected samples	This study did not involve samples collected from the field.
Ethics oversight	For invertebrate animals a study protocol is not needed according to local ethics oversight

Note that full information on the approval of the study protocol must also be provided in the manuscript.

Dual use research of concern

Policy information about dual use research of concern

Hazards

Could the accidental, deliberate or reckless misuse of agents or technologies generated in the work, or the application of information presented in the manuscript, pose a threat to:

No	Yes	
☒	☐	Public health
☒	☐	National security
☒	☐	Crops and/or livestock
☒	☐	Ecosystems
☒	☐	Any other significant area

Experiments of concern

Does the work involve any of these experiments of concern:

No	Yes	
☒	☐	Demonstrate how to render a vaccine ineffective
☒	☐	Confer resistance to therapeutically useful antibiotics or antiviral agents
☒	☐	Enhance the virulence of a pathogen or render a nonpathogen virulent
☒	☐	Increase transmissibility of a pathogen
☒	☐	Alter the host range of a pathogen
☒	☐	Enable evasion of diagnostic/detection modalities
☒	☐	Enable the weaponization of a biological agent or toxin
☒	☐	Any other potentially harmful combination of experiments and agents

ChIP-seq

Data deposition

☒ Confirm that both raw and final processed data have been deposited in a public database such as GEO.

☐ Confirm that you have deposited or provided access to graph files (e.g. BED files) for the called peaks.

Data access links<br>*May remain private before publication.*	The high-through sequencing data generated and analyzed during this study are available from NCBI at the following accession code: PRJNA763505
Files in database submission	We provide FASTQ files
Genome browser session<br>(e.g. UCSC)	FASTQ files can be convert to BigWig files, then BigWig files were visualized by Integrative Genomics Viewer (IGV).

Methodology

Replicates	Chip-seq data with three independent biological experiments.
Sequencing depth	Sequencing was single-ended. Each sample was sequenced to a depth at least 5 Million mapped reads.
Antibodies	H3K9me3 (Abcam, ab8898)
Peak calling parameters	Regions of IP enrichment over background were identified by the MACS2 (version 2.1.0) peak calling software (q-value threshold of 0.05 was used).
Data quality	edgeR was used to identify significant differentially expressed peaks between two groups using threshold as P value <0.05 and |log2 (fold change)|>2
Software	Bowtie2, MACS2, edgeR, ChIPseeker, DeepTools, R package.
